# TCBGY net for enhanced wear particle detection in ferrography using self attention and multi scale fusion

**DOI:** 10.1038/s41598-024-82961-z

**Published:** 2024-12-30

**Authors:** Lei He, Haijun Wei, Cunxun Sun

**Affiliations:** Merchant Marine College, Shanghai Maritime University, Shanghai, 201306 China

**Keywords:** Ferrography, Transformer, Small object detection, BiFPN, Model lightweigh, Engineering, Mechanical engineering

## Abstract

The intelligent identification of wear particles in ferrography is a critical bottleneck that hampers the development and widespread adoption of ferrography technology. To address challenges such as false detection, missed detection of small wear particles, difficulty in distinguishing overlapping and similar abrasions, and handling complex image backgrounds, this paper proposes an algorithm called TCBGY-Net for detecting wear particles in ferrography images. The proposed TCBGY-Net uses YOLOv5s as the backbone network, which is enhanced with several advanced modules to improve detection performance. Firstly, we integrate a Transformer module based on the self-attention mechanism with the C3 module at the end of the backbone network to form a C3TR module. This integration enhances the global feature extraction capability of the backbone network and improves its ability to detect small target wear particles. Secondly, we introduce the convolutional block attention module (CBAM) into the neck network to enhance salience for detecting wear particles while suppressing irrelevant information interference. Furthermore, multi-scale feature maps extracted by the backbone network are fed into the bidirectional feature pyramid network (BiFPN) for feature fusion to enhance the model’s ability to detect wear particle feature maps at different scales. Lastly, Ghost modules are introduced into both the backbone network and the neck network to reduce their complexity and improve detection speed. Experimental results demonstrate that TCBGY-Net achieves outstanding precision in detecting wear particles against complex backgrounds, with a *mAP@0.5* value of 98.3%, which is a 10.2% improvement over YOLOv5s. In addition, we conducted comprehensive ablation experiments, to validate the contribution of each module and the robustness of our model. TCBGY-Net also outperforms most current mainstream algorithms in terms of detection speed, with up to 89.2 FPS capability, thus providing favorable conditions for subsequent real-time online monitoring of changes in wear particles and fault diagnosis in ship power systems.

## Introduction

High reliability is the foundation for developing green and intelligent ships. Establishing a life prediction and fault diagnosis system for intelligent ship power units, conducting state monitoring research on early abnormal wear between friction pairs, and achieving real-time online state monitoring of crucial machinery and equipment are essential to ensure safe and reliable operation. Ferrography analysis technology, which examines wear particles in lubrication systems, extracts information such as the number, size, surface texture, shape, and color of wear particles to characterize the wear amount and mechanism^1^. This technology has become an effective basis for monitoring equipment wear status and health assessment and has been widely used in status monitoring, fault diagnosis, and preventive maintenance of ship power plants.

Traditional off-line ferrography analysis relies on expert experience to manually identify ferrography images, leading to low recognition precision and slow detection efficiency. With the introduction of image processing technology and artificial intelligence algorithms, the automation and intelligent development of ferrography analysis technology have become inevitable trends and research hotspots in wear status monitoring.The existing ferrography wear particle detection algorithms primarily include traditional image processing, machine learning, and deep learning methods. Traditional methods rely on preprocessing techniques such as filtering and edge detection, combined with feature extraction for wear particle detection. Machine learning algorithms detect particles by extracting features like HOG and SIFT and utilizing classifiers. In recent years, deep learning algorithms such as Faster R-CNN, YOLO, and SSD have shown excellent performance. These methods use convolutional neural networks to automatically extract features, enabling efficient and accurate detection of wear particles. Notably, YOLOv5, with its outstanding end-to-end detection capabilities and good generalization performance, has become a significant tool in wear particle detection.

Many scholars have studied this: Wang and colleagues^2^ proposed a two-layer classification model combining a BP neural network and CNN, increasing the recognition rate of fatigue and severe sliding wear particles to 85.7 and 80%. Peng^3^ proposed a hybrid convolutional neural network using transfer learning and a support vector machine for classifying common fatigue, oxidation, and spherical wear particles, increasing the classification precision by 23.27%. Fan^4^ proposed a virtual ferrography wear particle image intelligent recognition network based on AlexNet, effectively solving the problem of wear particle image recognition with small sample data by adding a regular term to suppress overfitting. Xie and others^5^ proposed a multi-channel coded convolutional neural network model (MCECNN), which improves the visible edges and surface features of wear particle images. However, its essence is just a tool for image enhancement and does not have object detection capabilities. Wang^6^ proposed a object detection algorithm based on an improved YOLOv4, solving the problem of recognition errors caused by the need for image segmentation in multi-wear image detection by traditional methods.

Nevertheless, these methods are complex for small target wear particles and image backgrounds, resulting in a high rate of false detection and missed detection, low recognition precision for overlapping and similar wear particles, and models that are too complex, requiring extensive time to train the neural network. The low detection precision in previous work is mainly due to the significant size variation of ferrography wear particles, making it difficult for existing algorithms to simultaneously detect both large and small particles. Additionally, complex backgrounds and noise interference increase the difficulty of detection, and deficiencies in feature extraction and multi-scale feature fusion further impact overall detection precision. These issues indicate the need for improvements in multi-scale feature fusion and background processing of wear particle images.

In recent years, the Transformer deep learning model based on the self-attention mechanism is a current research hotspot^7,8^. Fu^9^ proposed to improve the prediction head of YOLOv5s based on the Swin Transformer self-attention mechanism module to strengthen the network’s ability to extract multi-scale target features, but the detected mAP is only 68.5%. Bian^10^ combines the lightweight network EfficientDet with the Vision Transformer. In order to reduce the number of parameters and retain a large range of feature correlations, four 3 × 3 small convolutions are used to replace the 16 × 16 of the Embedding in the Vision Transformer. The convolutional layer effectively improves the efficiency and precision of object detection and recognition, and the real-time rate is 28FPS, which is relatively slow. Leng^11^ introduced the attention module into DETR and proposed a DETR-based Transformer structure model T-DETR for object detection. Unfortunately, the mAP on the COCO dataset is only 47.6%. The Transformer backbone network model has high computational complexity, is not suitable for use in resource-constrained environments, and the detection precision of ferrography images is low, while YOLOv5s uses a new network structure, which can achieve faster object detection speed and higher precision. Song^12^ and others proposed a lightweight object detection network of YOLOv5-MDC, which improved the original network by using the hybrid depth separable convolution and SE mechanism module, so that the detection precision of the network was better improved, and the model detection efficiency is high and the storage occupied is small. Gu^13^ proposed an improved object detection algorithm based on YOLOv5s. This method mainly uses the attention mechanism to improve the model’s ability to extract target features and detect low-quality targets. It studies the best embedding scheme and incorporates the idea of a soft threshold combined with the bilinear attention mechanism to achieve the purpose of alleviating the impact of low-quality data on detection precision of the model. Although YOLOv5s has an excellent performance in object detection, it still has the following shortcomings for the detection and recognition of small targets and ferrography wear particle images with complex surface textures:

1. The global feature information extraction ability of the backbone network is weak, and the ability to detect a small target, overlapping, and similar wear particle is weak;
2. There is too many small target wear particles in the ferrography wear particle image, the background is complex,the significant degree of wear particles are not high, and are easily disturbed;
3. When the feature maps of different scales are down-sampled and up-sampled multiple times, information will be lost, and the complexity of the model will be increased at the same time;
4. The detection speed is slow and cannot meet real-time requirements. Balancing speed and precision is a key challenge in wear particle detection. Real-time monitoring requires fast detection, while varying particle sizes and complex backgrounds demand precision.

In response to the above problems, this paper uses the YOLOv5s model as the framework and combines the advantages of the Transformer model of the self-attention mechanism to propose an improved YOLOv5s ferrography wear particle detection algorithm TCBGY-Net (Transformer Convolutional Block Attention Mechanism Ghost BiFPN-YOLOv5 Network). The proposed method addresses the limitations of existing wear particle detection by enhancing feature extraction, improving small and overlapping particle detection, and increasing efficiency with the C3TR, CBAM, BiFPN, and Ghost modules for better performance and reduced complexity. This paper optimizes the YOLOv5 architecture to improve precision with minimal impact on speed, achieving an effective balance in wear particle detection. The innovations of this paper are as follows:

1. Adopt the improved C3TR module to strengthen the global feature information extraction ability of the backbone network, expand the original detection scale at the same time, and improve the detection ability of small target wear particle, overlapping, and similar wear particle;
2. Introduce the CBAM attention mechanism to enhance the salience of the wear particle to be detected and suppress the interference of irrelevant information; and optimize the loss function to improve the convergence speed and positioning precision of the border regression, effectively solving the problem of overlapping wear particles.
3. Send the wear particle feature maps of different scales extracted by the backbone network to the BiFPN feature fusion network for multi-scale feature fusion, so that it can efficiently transfer information between different scale wear particle detection networks, and improve the detection of small target wear particle, identification precision of overlapping and similar wear particle while reducing the complexity of the network;
4. Introduce the Ghost module in the backbone network and the neck network, generate a large number of feature maps through simple linear operations, avoids redundant calculations while maintaining performance, realizes the lightweight of network, and improves detection speed.

## Self-designed ferrographic wear particle detection network TCBGY-Net

### Transformer convolutional block attention mechanism ghost BiFPN-YOLOv5 network (TCBGY-Net) for wear particle detection

**Fig. 1:**
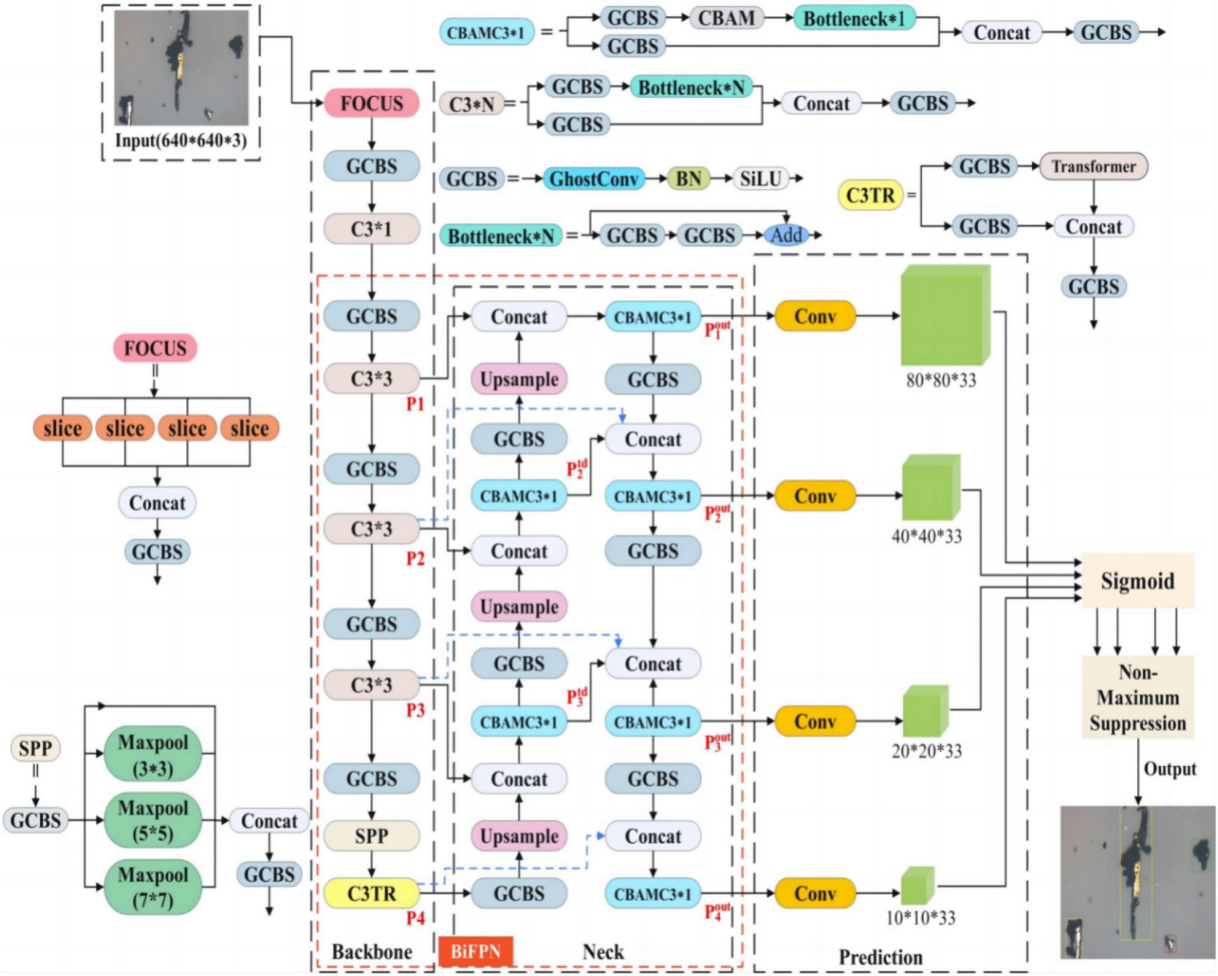
Architecture of the TCBGY-Net: Transformer-based YOLOv5 with convolutional block attention mechanism, ghost module, and BiFPN for wear particle detection.

This paper presents TCBGY-Net, a new ferrography wear particle detection algorithm based on a self-attention mechanism and multi-scale feature fusion, developed through the optimization of the network architecture, as shown in (Fig. 1). Input the generated 4 feature maps into the prediction network, use the convolutional layer with an activation function to adjust the number of channels to *C*, and limit the prediction between 0 and 1, through AutoML^14^ determines the value of the number of channels as 33, and the final convolutional layer prediction tensor is *N* × *N* × 33 (where N is 10, 20, 40 or 80). The four prediction branches are responsible for detecting wear particles of different sizes, among which the deepest 10 × 10 × 33 feature maps are suitable for detecting large wear particles, 80 × 80 × 33 feature maps are suitable for predicting small wear particles, and 20 × 20 × 33 and 40 × 40 × 33 feature maps are suitable for detecting medium-sized wear particles. Finally, the NMS algorithm is employed to remove redundant and low-quality predicted bounding boxes and provide the best predicted results for each target wear particle.

### Introducing the improved C3TR module

**Fig. 2:**
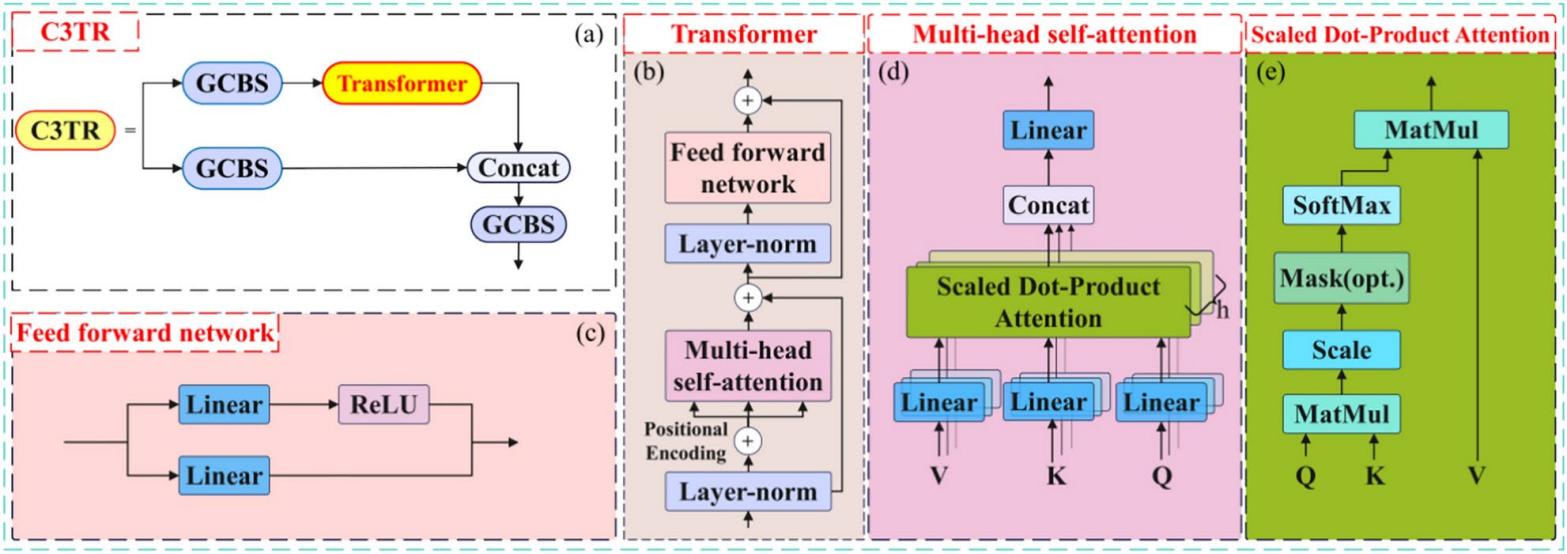
Structure of the improved C3 Module with transformer encoder (C3TR).

The core of the Transformer is the multi-head attention mechanism^15^, which has a global receptive field^16^. The structure consists of an encoder and a decoder. This paper utilizes the Transformer encoder structure. For small targets, deep convolution is typically employed to reveal their features. While the Bottleneck module in YOLOv5 performs well in image classification tasks and can extract low-level features such as edges, textures, and colors from wear particle images, it does not perform as effectively as the Transformer module in extracting high-level features like semantic information and overall characteristics of wear particle images. The Transformer module, through its self-attention and multi-head attention mechanisms, excels in extracting semantic information, leading to more accurate recognition^17^. Additionally, the Transformer module offers better parallelism and scalability, enabling efficient utilization of hardware resources like GPUs for accelerated model training and inference. In this paper, the method replaces the Bottleneck in the original YOLOv5s’ C3 module with the Transformer encoder structure, forming a new C3TR module. This addition to the last C3 module in the YOLOv5s backbone network enhances the capture and detection of small targets, overlapping particles, and similar wear particles, improving model performance and computational efficiency. The structure is depicted in (Fig. 2).

Explanation: Fig. 2a illustrates the C3TR module structure; Fig. 2b depicts the Transformer module within the C3TR module; Fig. 2c shows the feed-forward network within the Transformer module; Fig. 2d displays the Multi-head self-attention structure in the Transformer module; and Fig. 2e presents the Scaled Dot-Product Attention module within the Multi-head self-attention module.

The self-attention used in the Transformer network is a normalized scaled dot product attention mechanism, and the calculation expression is as in Eq. (1):

1 $$Attention\left( {Q,K,V} \right) = softmax\left( {\frac{{QK^{T} }}{{\sqrt {d_{K} } }}} \right)V$$

In the equation, *Q, K,* and *V* represent the query vector, key vector, and value vector respectively, and are dimensions of input features. As per Eq. (1), initially, a transposition operation is performed on *K*. Following this, a matrix product operation is conducted between the transposed *Q* and *K* to derive a vector. This vector is then scaled using a scaling factor. Subsequently, an activation calculation is applied to the obtained matrix using a specific function. The resultant matrix represents the correlation between each pixel and other pixels, referred to as the attention score matrix. This matrix is then applied to *V*, leading to the fusion of the eigenvector value of each pixel in the matrix with other pixels, compared to the features of the input matrix^18^. In the Transformer encoder, the multi-head self-attention mechanism employs *h* scaled dot product attention mechanisms in parallel, thereby dividing the input feature matrix into *h* independent attention heads. Without necessitating additional computations, multi-head attention enhances the diversity of the feature subspace, improving the algorithm’s capacity to extract information from different positions. Its calculation is depicted in Eq. (2).

2 $$\begin{gathered} MultiHead\left( {Q,K,V} \right) = Concat\left( {head_{1} ,...,head_{h} } \right)W^{O} \hfill \\ where\begin{array}{*{20}c} {} & {} \\ \end{array} head_{i} = Attention\left( {QW_{i}^{Q} ,KW_{i}^{K} ,VW_{i}^{V} } \right) \hfill \\ \end{gathered}$$

where: $W_{i}^{Q} \in R^{{d_{model} \times d_{Q} }}$,$W_{i}^{K} \in R^{{d_{model} \times d_{K} }}$,$W_{i}^{V} \in R^{{d_{model} \times d_{V} }}$,$W^{O} \in R^{{d_{model} \times hd_{V} }}$ are parameter matrices, $d_{model}$ are vector dimensions,$d_{Q}$,$d_{K}$,$d_{V}$ are input feature dimensions.

### Introduce the improved CBAMC3 × 1 module

**Fig. 3:**
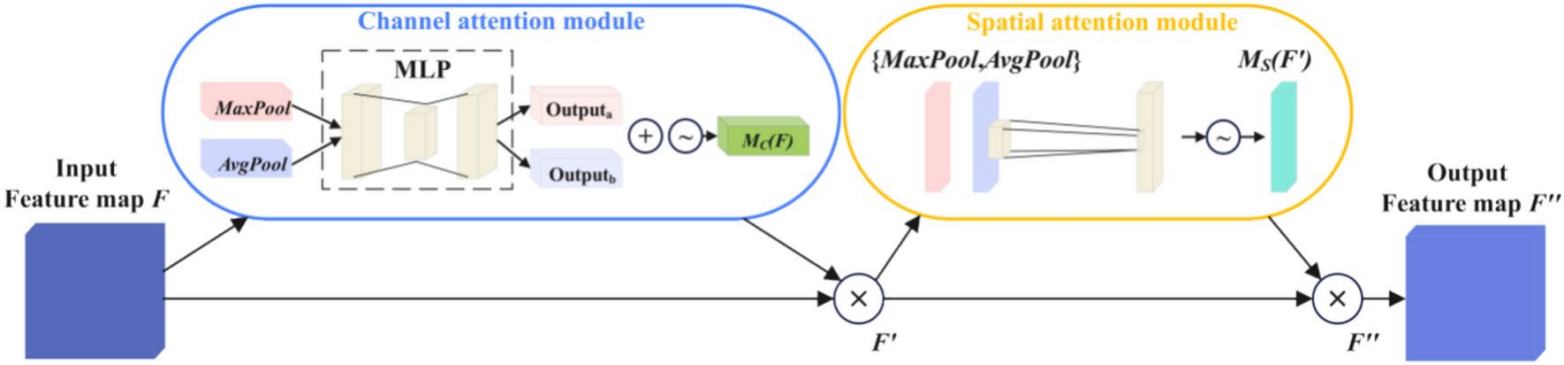
Structure of the convolutional block attention mechanism (CBAM) module for enhanced feature attention.

In order to solve the problem of low conspicuousness of wear particles caused by complex backgrounds, CBAM (Convolutional Block Attention Module) was introduced into the C3 module of YOLOv5s^19,20^. Its structure is shown in Fig. 3, where *Mc* and *Ms* represent channel attention modules respectively, spatial attention modules.

Given a feature map $F = R^{C \times H \times W}$, *C* is the number of input feature channels, $H \times W$ is the size of the input feature map, and *R* is a three-dimensional matrix composed of *C*, *H*, and *W*, the calculation process is as in Eq. (3), where $\otimes$ represents the matrix between Multiply elements at corresponding positions.

3 $$\begin{gathered} F^{\prime} = M_{C} \left( F \right) \otimes F \hfill \\ F^{\prime\prime} = M_{S} \left( {F^{\prime}} \right) \otimes F^{\prime} \hfill \\ \end{gathered}$$

The CBAM module first sends *F* to the channel attention module, obtains the information of each channel through *AvgPool* and *MaxPool*, and superimposes the obtained parameters through an MLP, and then after activation by the $sigmoid$ function, the channel attention feature $M_{C} \left( F \right)$ is obtained, and its calculation is as in Eq. (4):

4 $$\begin{gathered} M_{C} \left( F \right) = \sigma \left( {MLP\left( {Avgpool\left( F \right)} \right) + MLP\left( {Maxpool\left( F \right)} \right)} \right) \\ = \sigma \left( {W_{1} \left( {W_{0} \left( {F_{Avg}^{C} } \right)} \right) + W_{1} \left( {W_{0} \left( {F_{Max}^{C} } \right)} \right)} \right) \\ \end{gathered}$$

In the equation: $\sigma$ represents the $sigmoid$ nonlinear activation function; $W_{0} \in R^{{C/\left( {r * C} \right)}}$, $W_{1} \in R^{{\left( {C * C} \right)/r}}$, $W_{0}$ represent the weight of the first layer of multi-layer perceptron; $W_{1}$ is the weight of the second layer. $F_{Avg}^{C}$ and $F_{{M{\text{a}}x}}^{C}$ represent the global *AvgPool* and *MaxPool* operations of the channel attention mechanism respectively, and $r$ is the reduction rate.

After sending the given feature map $F^{\prime}$ into the spatial attention module, the spatial information is gathered along the channel dimension through *AvgPool* and *MaxPool* to generate a spatial feature map, and then convolution of $1 \times 1$ and activation of the $sigmoid$ function are used to obtain the spatial attention feature; and then multiply element by element with $F^{\prime}$ to finally get the spatial attention feature map $M_{S} \left( {F^{\prime}} \right)$ . Its calculation is as Eq. (5):

5 $$\begin{gathered} M_{S} \left( {F^{\prime}} \right) = \sigma \left( {f^{7 * 7} \left( {Avgpool\left( {F^{\prime}} \right);Maxpool\left( {F^{\prime}} \right)} \right)} \right) \\ = \sigma \left( {f^{7 * 7} \left( {F_{Avg}^{S} ;F_{Max}^{S} } \right)} \right) \\ \end{gathered}$$

In the equation: $F_{{A{\text{vg}}}}^{S}$, $F_{{M{\text{ax}}}}^{S}$ represents the global *AvgPool* and *MaxPool* operations in the spatial attention mechanism; $f^{7 \times 7}$ represents the convolution operation of size $7 \times 7$.

In order to study the impact of the position of CBAM module addition on the detection performance of the algorithm, we fused the CBAM module in the backbone network of YOLOv5s and the C3 module of the neck network to form the CBAMC3 × N module, and used the dataset mentioned in section "Preparation of wear particle dataset and enhancement of loss function" and training parameters. The training results show that the *mAP@0.5* of Backbone + CBAM is 91.2%, and the *mAP@0.5* of Neck + CBAM is 92.7%. Therefore, we choose to add the CBAM attention mechanism to the C3 module in the Neck network.

### Introducing the improved BiFPN structure

**Fig. 4:**
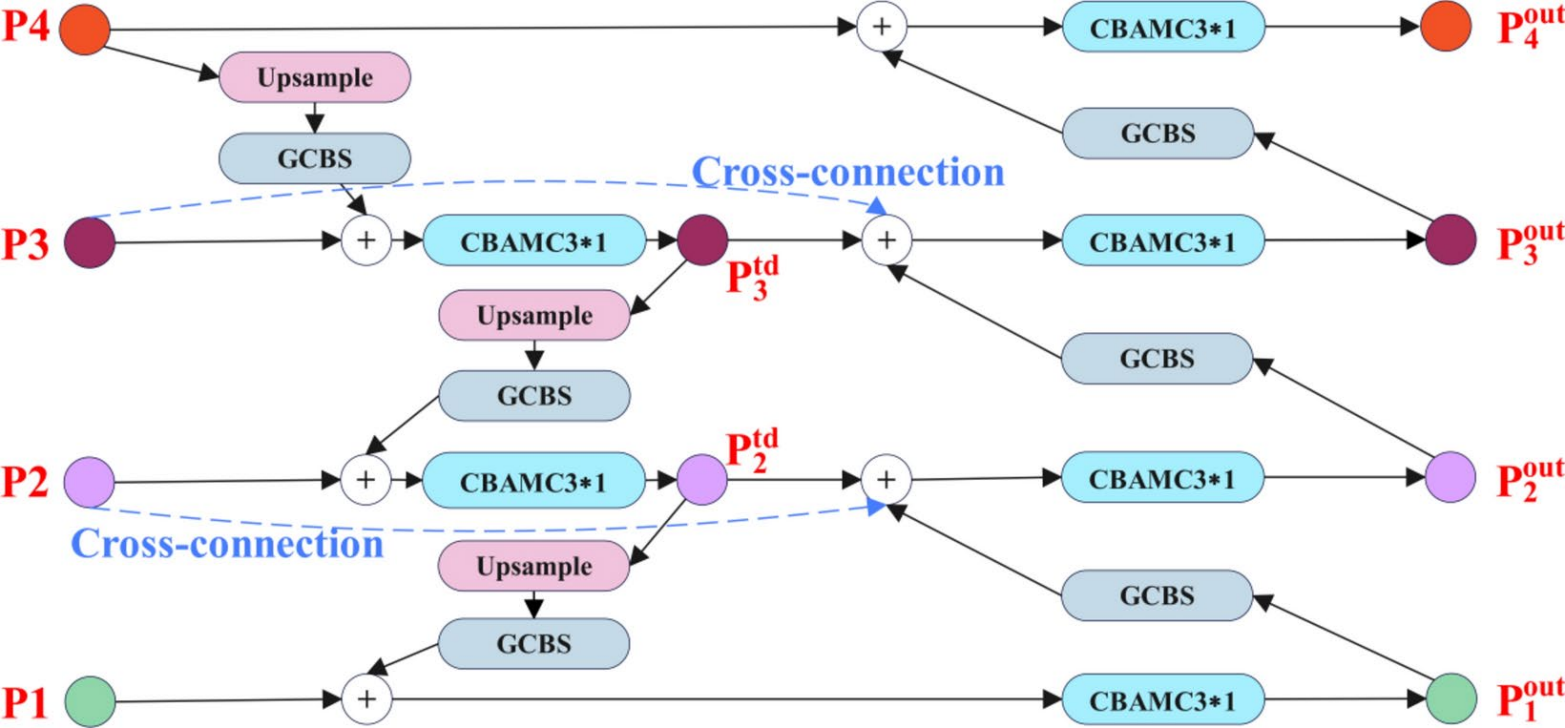
Structure of the improved bidirectional feature pyramid network (BiFPN) in TCBGY-net for enhanced multi-scale feature fusion.

In general, a feature map with a larger scale is more suitable for detecting small target wear particles, while a feature map with a smaller scale is more appropriate for detecting large target wear particles. A feature pyramid that combines both can better detect both large and small target wear particles. To enhance the robustness of multi-size wear particle representation, a residual module is embedded within BiFPN^21^ to aggregate these feature maps P1-P4 at different resolution scales. Unlike the traditional top-down FPN^22^ and PANet^23^, BiFPN treats each bidirectional path as a feature network layer and performs multiple calculations on the parameters of the same layer to achieve more feature fusion. Figure 4 presents a simplified bidirectional feature fusion network.

*P*_*4*_ to *P*_*1*_ is a top-down path of aggregated feature maps, which can preserve rich semantic information for wear particle classification. This top-down path adopts the GCBS module and up-sampling to adjust the resolution and channels of the high-level feature maps to be consistent with those of the low-level feature maps while implementing in this path with the improved CBAMC3 × 1 module to achieve more advanced feature map fusion. Since some nodes with only one input edge contribute less to feature fusion, the intermediate nodes of *P*_*4*_ and *P*_*1*_ are deleted to simplify the network structure. The bottom-up aggregation path is from ${P}_{1}^{out}$ to ${P}_{4}^{out}$. In this path, low-level feature maps go through the GCBS module and down-sampling and are fused with feature maps of intermediate nodes and input nodes. Meanwhile, the improved CBAMC3 × 1 module is also adopted to achieve more advanced feature map fusion in the bottom-up path. This path greatly shortens the information path from low-level to high-level and can make good use of the position information in low-level feature maps to improve the localization of wear particles^24^.

Different from traditional feature fusion methods, the enhanced BiFPN also distinguishes the fusion of various input features to discern the significance of each input. It employs a weighted fusion mechanism. The upgraded BiFPN utilizes a rapid normalization technique, outlined as follows:

6 $$Out = \sum\limits_{i = 1}^{M} {\frac{wi}{{\varepsilon + \sum {_{i = 1}^{M} wi} }}I} i$$

where *w*_*i*_ is learnable weights, *I*_*i*_ is the *i*-th input feature map, *M* is the total number of input feature maps, and ε = 0.0001 is a parameter to avoid numerical instability. The intermediate feature map ${P}_{3}^{td}$ on the top-down path and the output feature map ${P}_{3}^{out}$ on the bottom-up path can be expressed in the following way:

7 $$P_{3}^{{{\text{td}}}} = \left( {CBAMC3 \times 1} \right) \otimes \left( {\frac{{w_{1} \times P_{3} + w_{2} \times GCBS\_up\left( {P_{4} } \right)}}{{w_{1} + w_{2} + \varepsilon }}} \right)$$

8 $$P_{3}^{{{\text{out}}}} = \left( {CBAMC3 \times 1} \right) \otimes \left( {\frac{{w^{\prime}_{1} \times P_{3} + w^{\prime}_{2} \times P_{3}^{{{\text{td}}}} + w^{\prime}_{3} \times GCBS\left( {P_{2}^{{{\text{out}}}} } \right)}}{{w^{\prime}_{1} + w^{\prime}_{2} + w^{\prime}_{3} + \varepsilon }}} \right)$$

Among them, GCBS_up is a convolution operation + up sampling operation, and GCBS is just a convolution operation for down sampling and feature processing, and the rest of the features are constructed in a similar manner.

Finally, the improved BiFPN feature fusion module can generate different levels of multi-scale output feature maps (${P}_{4}^{out}$, ${P}_{3}^{out}$, ${P}_{2}^{out}$ and ${P}_{1}^{out}$). These output feature maps integrate the position information in the low-level features with the richer semantic information in the high-level features through convolution operations, up sampling operations, and residual operations, which is conducive to the accurate positioning of wear particles of different sizes.

### Introducing the improved GCBS module

In order to meet the real-time requirements of online wear particle detection, this paper introduces Ghost convolution to establish a lightweight TCBGY-Net network from the perspective of reducing network complexity and model calculation^25^. Compared with ordinary convolution, Ghost convolution uses a simple and easy-to-operate linear transformation to replace some ordinary convolutions to generate a large number of feature maps, and mines information from original features at a small computational cost^26^. It is a lightweight and efficient method, namely Plug and play modules^27^. The operation of ordinary convolution is Eq. (9):

9 $$Y = X \times f + b$$

where:*X* is the input image, × represents the convolution operation, *Y* is the output feature map, and *b* is the bias term.

**Fig. 5:**
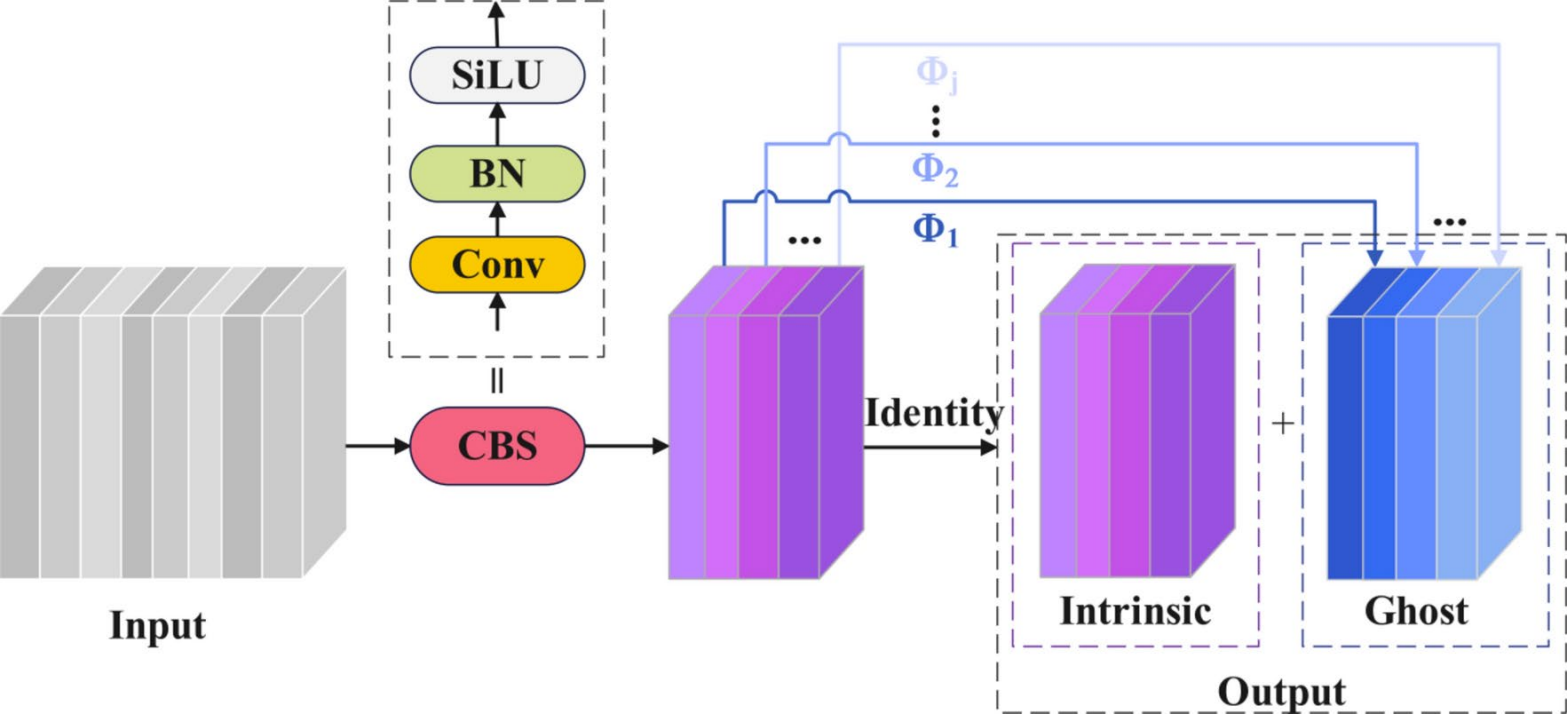
The implementation principle of Ghost Convolution for reducing computational complexity.

The working principle of Ghost convolution is shown in Fig. 5. The specific implementation steps are as follows:

1) Firstly, use a small number of ordinary convolutions to generate a small number of input image feature maps, as shown in Eq. (10):

10 $$Y^{\prime} = X \times f^{\prime}$$

where:$f^{\prime}$ is an ordinary convolution filter, and $Y^{\prime}$ is to generate a feature map. For the convenience of calculation, the bias term *b* is omitted here.

2) Use linear transformation to generate a large number of redundant feature maps, that is, Ghost feature maps, as shown in Eq. (11):

11 $$y_{ij} = \phi_{j} \left( {y^{\prime}_{i} } \right)\;\;\forall i = 1,...,m\;\;\forall j = 1,...,s$$

In the formula: ${\text{y}}_{ij}$ is the final generated Ghost feature map, ${\text{y}}_{{\text{i}}}^{\prime }$ is the Ith intrinsic feature mapping of $Y^{\prime}$; $\varphi_{{\text{j}}}$ is the *j*th linear operation and is used to generate the *j*th mapping.

(1) Finally, a small number of feature maps generated by ordinary convolution are stitched together with Ghost feature maps to form the final feature map.

## Preparation of wear particle dataset and enhancement of loss function

In order to verify the effectiveness of the method proposed in this paper, an experiment was carried out under pytorch, and the configuration is as follows: the operating system is Windows10, 64-bit, 16G memory, the CPU is Intel(R) Core(TM) i9-10920X@3.50 GHz, and the GPU is NVIDIA GeForce RTX3090Ti.

### Production of an experimental data set

**Fig. 6:**
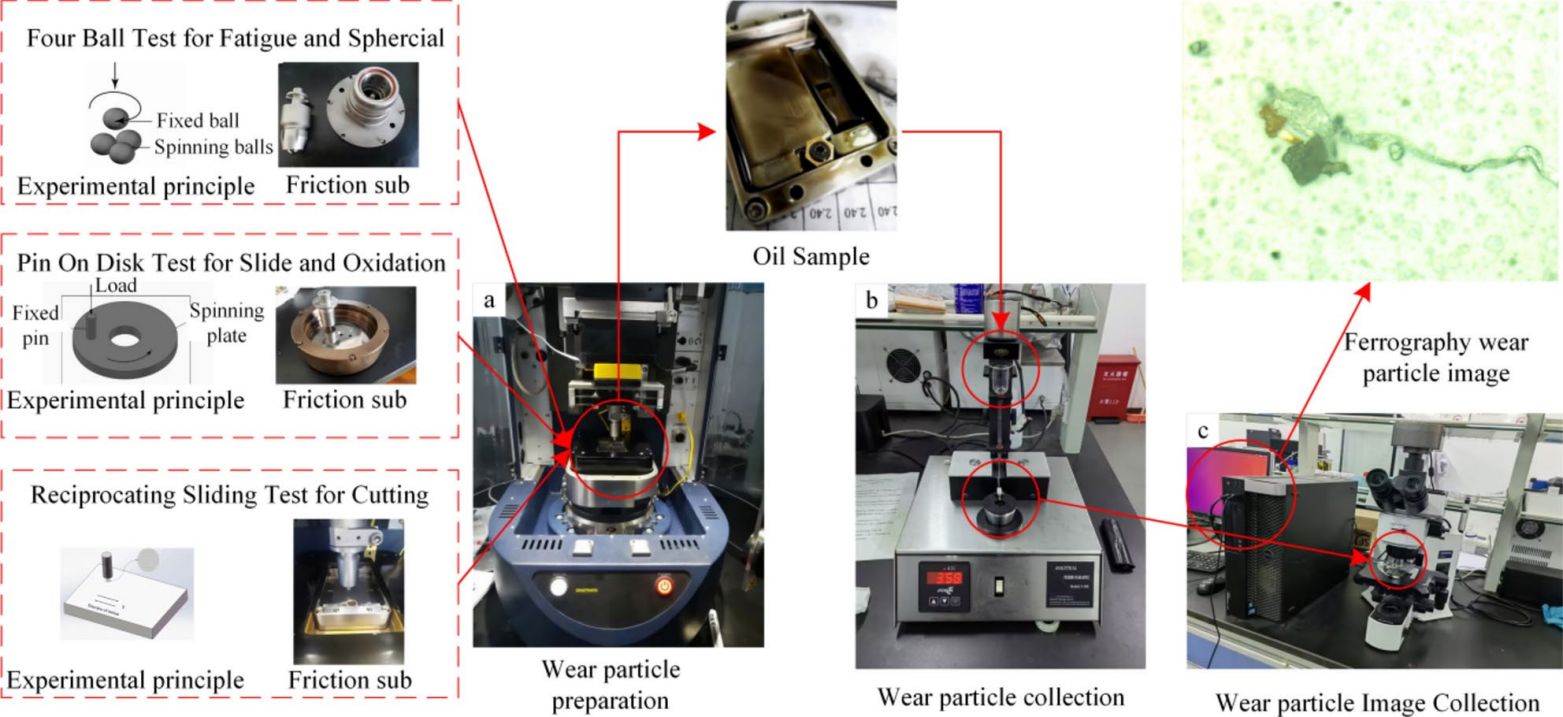
Dataset production flow chart.

The wear particle image data set required for the experiment is produced by the BRUKER friction and wear testing machine, and its model is UMT Tribo Lab, as shown in (Fig. 6a). This equipment produces the required wear particle through four-ball, pin-on-disc, and reciprocating sliding tests, and records important parameters such as load, displacement, temperature, friction force, friction coefficient, and wear amount in the friction test in real time through sensors and control software. Table 1 shows the main parameters of the BRUKER friction and wear testing machine.

**Table 1:** Main parameters of BRUKER friction and wear testing machine.

Parameter	Control range
Load force range	0.1mN ~ 1000N
Loading force precision	< 0.1%
Maximum friction	500N
Reciprocating modules	≤ 120mm
Reciprocating motion rate	0.001 ~ 100mm/s
Temperature control range	− 35 ~ 1000℃
Temperature control precision	< 0.1℃

The prepared wear particle was made into spectrograms by an analytical spectrometer (Fig. 6b), and then the pictures of the original wear particle were obtained by taking pictures with a microscope observer (Fig. 6c). Because the size of the pictures taken by the optical microscope used in the experiment is 2568 × 1912, the resolution is too high, and it is necessary to rotate and crop these original wear particle pictures, and use OpenCV for data augmentation to obtain 2500 pictures of 640 × 640 wear particle, where there is multiple wear particle in one wear particle picture. The specific production process is shown in (Fig. 6).

**Fig. 7:**
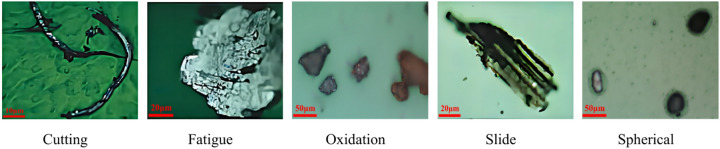
Various detected wear particle images.

**Fig. 8:**
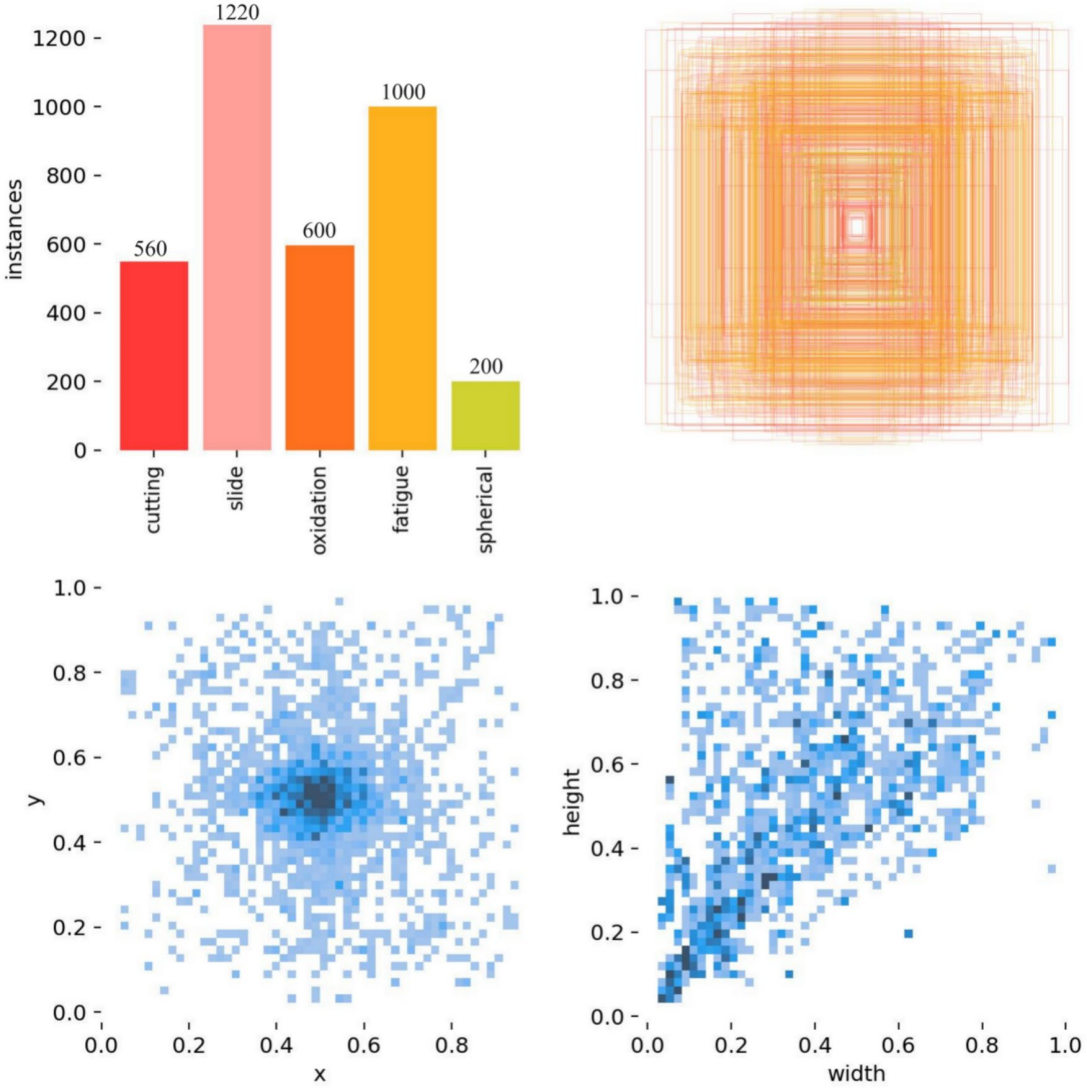
Detection label results.

Five types of wear particles were obtained in the experiment, namely Spherical, Severe Slide, Fatigue, Oxidation, and Cutting, as shown in (Fig. 7). Then use the LabelImg labeling tool to mark these pictures and organize them into the COCO dataset format. Finally, the obtained data set is divided according to the ratio of training set, verification set, and test set at 6:2:2, and is detected through the training parameters set in section "Training parameter setting". Given that the dataset was manually created, we implemented measures to mitigate the risk of overfitting, including data augmentation techniques such as random cropping, rotation, and flipping to enhance the diversity of the training data. Additionally, k-fold cross-validation was employed to ensure the stability and generalizability of the model’s performance.The detected label results are shown in (Fig. 8). The download link for the wear particle image dataset is https://github.com/helei0014shmtu/Ferrographic-wear-particle- image-dataset/releases/tag/V1.0.0.

Figure 8 presents a comprehensive analysis of various data characteristics in the wear particle detection experimental dataset, including the frequency of different types of wear particles, the hierarchical structure of feature fusion, the spatial distribution of wear particle feature points, and the correlation between the width and height of wear particle bounding boxes. The bar chart shows that the “slide” and “fatigue” datasets have the highest quantities, while “spherical” is the lowest. The nested rectangle diagram illustrates the relationships in feature fusion or attention mechanisms. The two scatter plots respectively show the concentration areas of feature distribution and the linear relationship between width and height. These visualizations provide important references for understanding the characteristics of wear particles and improving detection algorithms.

### Training parameter setting

During the training process, the number of iterations is set to 200 rounds. The initial learning rate and the termination learning rate are both set to 0.01, as these values strike a balance between convergence speed and stability, allowing the model to learn effectively without significant oscillations. The choice of 200 iterations was made to ensure sufficient training for convergence while preventing overfitting. The learning rate of 0.01 was determined through a systematic hyperparameter tuning process, including grid search and cross-validation, which demonstrated that it provides an optimal balance between convergence speed and model stability, enabling efficient gradient updates while minimizing the risk of divergence. The batch size is set to 32 to balance between memory efficiency and stable gradient estimation. A larger batch size would require significantly more memory, while a smaller batch size could lead to unstable gradient updates.

The momentum value of 0.937 is used to help accelerate gradient vectors in the right direction, leading to faster convergence. The weight decay of 0.0005 is applied to prevent overfitting by adding a regularization term, which helps in maintaining generalization capability. To speed up model training, this paper utilizes transfer learning by using the trained model weights on the dataset as the pre-training weights for the TCBGY-Net model^28^. Transfer learning is particularly effective in this context as it allows the model to leverage previously learned features, thereby reducing the training time and improving convergence, especially when dealing with limited labeled data. Some hyperparameter settings during network training are shown in (Table 2).

**Table 2:** Training hyperparameter settings.

Configuration parameters	Batch size	Epoch	Initial learning rate	Final learning rate	Momentum	Weight decay
Values	32	200	0.01	0.01	0.937	0.0005

### Loss function of TCBGY-Net

In this paper, the confidence loss *L*_*obj*_, classification loss *L*_*cls*_ and positioning loss *L*_*box*_ are used to guide and train the model, and the final loss function is obtained by summing the confidence loss, classification loss, and positioning loss, as shown in Eq. (12):

12 $$L = \lambda_{obj} L_{obj} + \lambda_{cls} L_{cls} + \lambda_{box} L_{box}$$

where $\lambda_{obj}$,$\lambda_{{{\text{cls}}}}$ and $\lambda_{{{\text{box}}}}$ are the scaling factors of the three corresponding losses, because, in the overall loss function, the three category losses in the model contribute differently to the overall loss, so in this paper the adaptive weighting algorithm^29^ is used and passed Experimental training results in different scaling factors with values of 1, 0.5 and 0.1. In this paper, the binary cross-entropy loss function^30^ is used to calculate the classification loss and confidence loss, and the *EIoU* loss^31^ is used to calculate the positioning loss. The *EIoU* loss divides the loss function into three parts: the overlapping loss $L_{IoU}$ of the predicted frame and the real frame, center distance loss $L_{dis}$ and width and height loss $L_{{{\text{asp}}}}$. Each loss is expressed by the following equation:

13 $$\begin{gathered} L_{obj} = - \sum\limits_{i = 1}^{N*N} {\sum\limits_{j = 1}^{3} {I_{ij}^{obj} } } \left[ {C_{ij} \ln \left( {C_{ij}^{*} } \right) + \left( {1 - C_{ij} } \right)\ln \left( {1 - C_{ij}^{*} } \right)} \right] - \\ \lambda_{noobj} \sum\limits_{i = 1}^{N*N} {\sum\limits_{j = 1}^{3} {I_{ij}^{noobj} } } \left[ {C_{ij} \ln \left( {C_{ij}^{*} } \right) + \left( {1 - C_{ij} } \right)\ln \left( {1 - C_{ij}^{*} } \right)} \right] \\ \end{gathered}$$

14 $$Lcls = - \sum\limits_{i = 1}^{N*N} {I_{ij}^{obj} \sum\limits_{c \in cls} {\left\{ {Pij\left( c \right)\ln \left[ {P_{ij}^{*} \left( c \right)} \right] + \left[ {1 - Pij\left( c \right)} \right]\ln \left[ {1 - P_{ij}^{*} \left( c \right)} \right]} \right\}} }$$

15 $$LEIoU = LIoU + Ldis + Lasp = 1 - IoU + \frac{{\rho^{2} \left( {b,bgt} \right)}}{{c^{2} }} + \frac{{\rho^{2} \left( {w,wgt} \right)}}{{c_{w}^{2} }} + \frac{{\rho^{2} \left( {h,hgt} \right)}}{{c_{h}^{2} }}$$

In the equation, *N* indicates that the feature map of the last output of the network is divided into $N \times N$ grids; $I_{ij}^{obj}$ indicates the anchor box with the target; $I_{ij}^{noobj}$ indicates the anchor box without the target; $\lambda noobj$ indicates the confidence loss weight coefficient of the anchor box without the target, which is set to 0.5; $C_{ij}^{*}$ and $Cij$ are the predicted value and the real value of the bounding box confidence respectively; $P_{ij}^{*}$ and $Pij$ are the predicted and true value of the *j*th bounding box predictor variable in the *i* grid cell; *IoU* is the ratio of the area of the intersection of the prediction box and the real box to the area of the merger, ρ is the Euclidean distance between the center points of the two boxes, *c* is the diagonal length of the smallest enclosing box covering the two boxes, where *w* and *h* are the widths of the ground truth boxes, respectively and height, $w_{gt}$ and ${\text{h}}_{gt}$ are the width and height of the prediction box, respectively.

To sum up, the total loss $L_{{{\text{to}}tal}}$ of the network is defined as the average loss on the *N* prediction branches, and the calculation equation is as follows:

16 $$\begin{gathered} Ltotal = \frac{1}{N}\sum\limits_{i = 1}^{N} {L_{n} } \hfill \\ L_{n} = - w\left[ {yn \times \ln xn + \left( {1 - yn} \right) \times (1 - \ln xn)} \right] \hfill \\ \end{gathered}$$

In the equation: *N* represents the number of samples; $L_{{\text{n}}}$ is the loss corresponding to the *n*th sample, ${x}_{n}$ is the predicted probability of the corresponding sample, which is activated by $sigmoid$,${\text{y}}_{n}$ is the real probability of the category of the corresponding sample, the value is 0 or 1, and $\omega$ is the super parameter, representing the true class of the sample. The $sigmoid$ function is shown in Eq. (17):

17 $$sigmoid = \frac{1}{{1 + e^{( - x)} }}$$

## Results and analysis

### Experimental evaluation index

In order to objectively evaluate the advantages of the algorithm in this paper, the experiment uses precision(*P*), recall(*R*), Average Precision(*AP*), *mAP@0.5* $,{F}_{1}$ as the evaluation indicators of model performance, and selects FPS (Frames Per Second) is used as the model speed evaluation index, and the calculation equations of each index are as follows:

18 $$P = \frac{NTP}{{NTP + NFP}}$$

19 $$R = \frac{NTP}{{NTP + NFN}}$$

20 $$AP = \int_{0}^{1} {P\left( R \right)} dR$$

21 $$F_{1} = \frac{2 \times P \times R}{{P + R}}$$

22 $$mAP = \frac{{\sum\limits_{i = 1}^{N} {APi} }}{N}$$

23 $$FPS = \frac{1}{taverage}$$

Among them, *P* represents the precision rate of whether all detected positive samples in a certain category are actual positive samples, and *R* represents the proportion of actual positive samples that are predicted to be positive samples. *TP* represents the number of positive samples that are correctly predicted, *FP* represents the number of positive samples that are incorrectly predicted, *FN* represents the number of negative samples that are incorrectly predicted, and *N* represents the number of detection categories. *mAP@0.5* means when the *IOU* is 0.5, the average *AP* of all single categories, where *AP* is the area enclosed under the *P-R* curve. FPS indicates the number of frames transmitted per second, where taverage is the average time to detect a picture.

### Analysis of experimental results

**Fig. 9:**
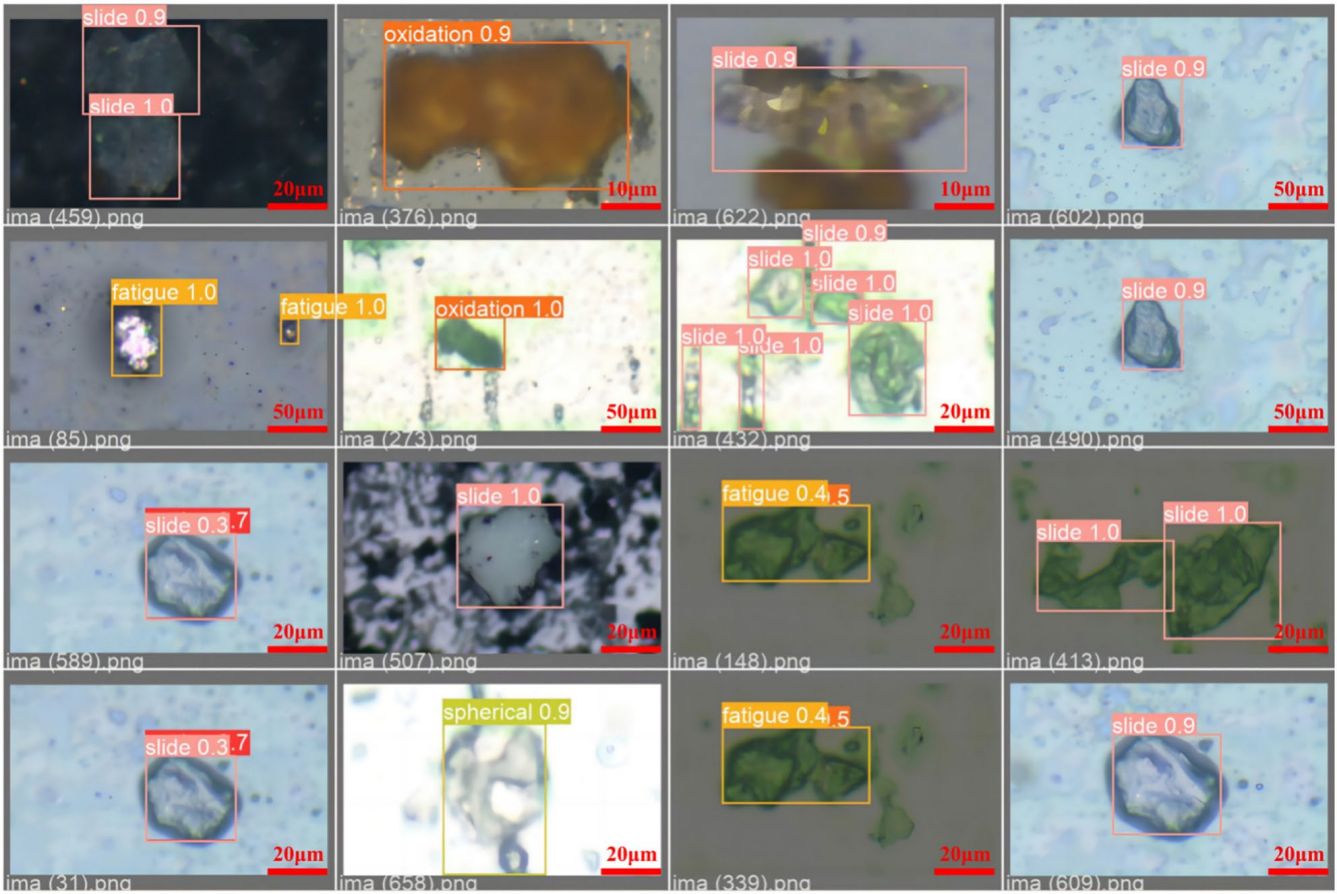
TCBGY-Net training detection results for some wear particles.

**Fig. 10:**
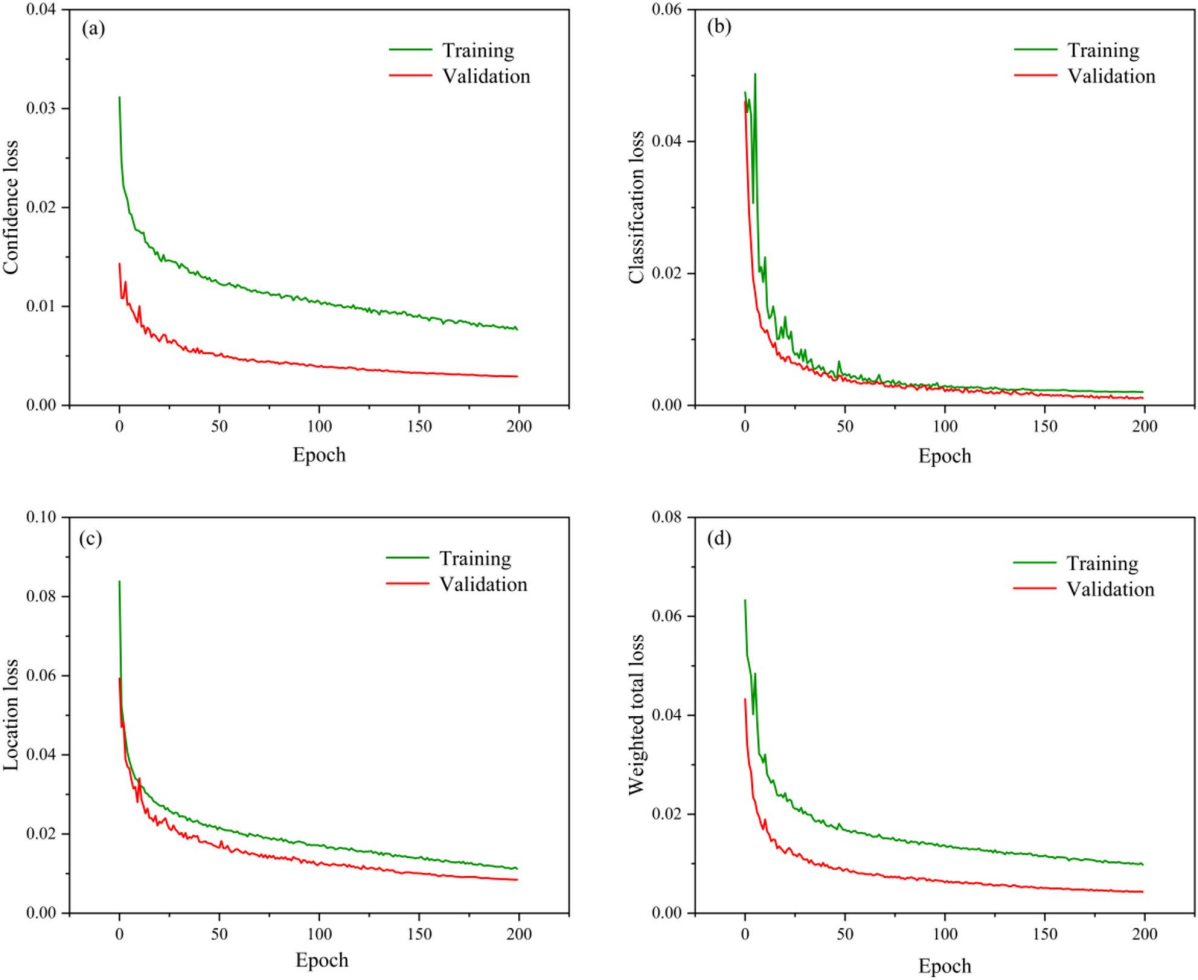
Loss function change curve: (**a**) Confidence loss curve; (**b**) Classification loss curve; (**c**) Location loss curve; (**d**) Weighted total loss curve.

In this paper, we use the data set and training parameters provided in Section "Preparation of wear particle dataset and enhancement of loss function" for training. Part of the training and detection results are shown in Fig. 9, and the loss function curves of the training set and the verification set are recorded respectively. The loss function includes Confidence loss, Classification loss, Location loss and Weighted total loss, as shown in Fig. 10. As the number of iterations increases, the training errors of all categories show a downward trend, and the network converges faster in the initial stage. The validation loss oscillates to some extent around 15 epochs, but after sufficient training, the training and validation losses eventually stabilize. The total training loss and total validation loss finally stabilized around 0.00978 and 0.00432 after 200 epochs.

**Fig. 11:**
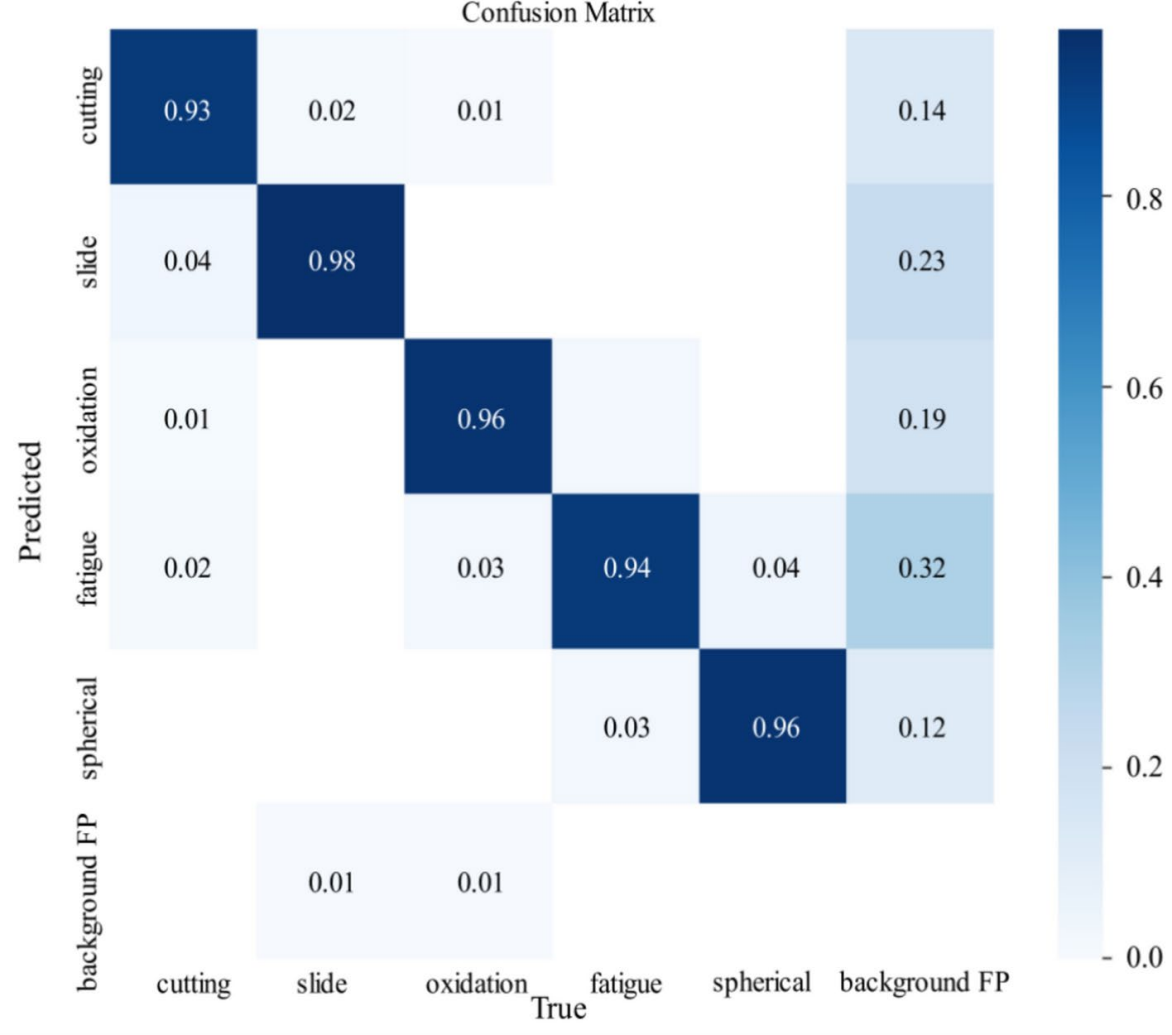
Confusion matrix.

**Fig. 12:**
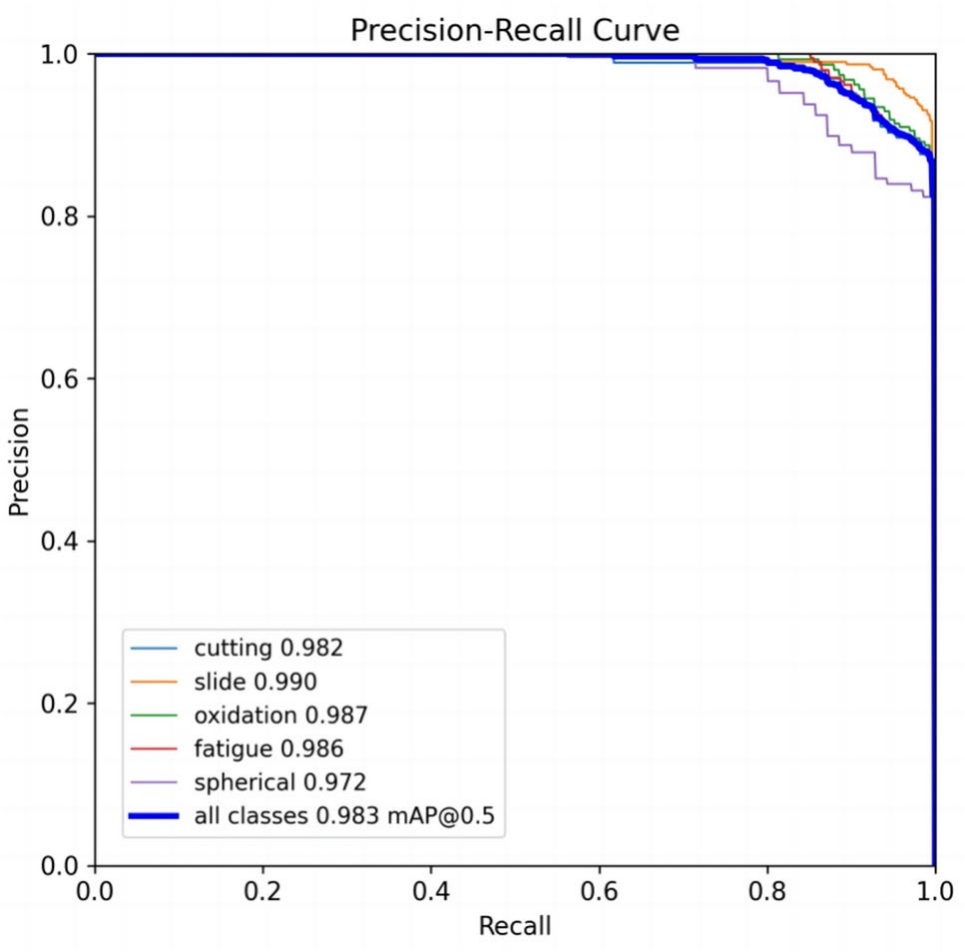
PR curve.

**Fig. 13:**
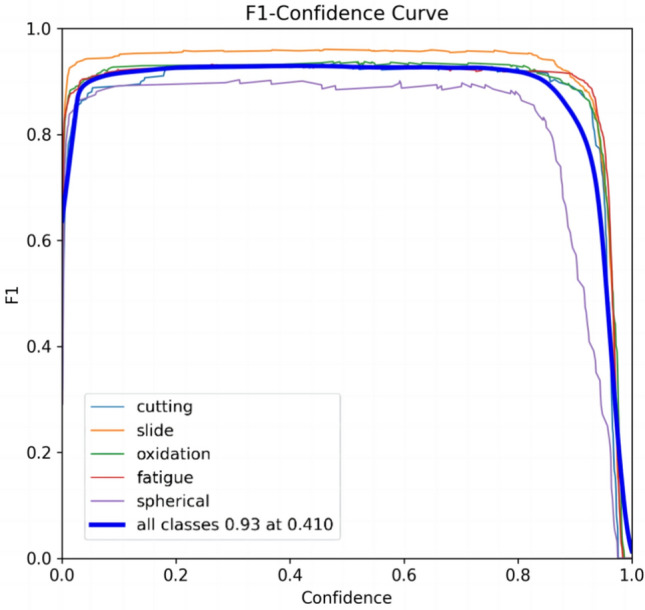
*F1* curve.

In order to more intuitively evaluate the precision of TCBGY-Net for five different wear particle detection and identification tasks, the changes in the classification precision of the five types of wear particles can be seen through the confusion matrix, where the slide is 0.98 and the recognition precision is the highest, and the lowest is cutting also reached 0.93, as shown in (Fig. 11). Through the *P-R* curve, we can see the change of the *P-R* value of the five abrasive grains, among which the cutting is 0.982, the slide is 0.990, the oxidation is 0.987, the fatigue is 0.986, the spherical is 0.972, and all classes *map@0.5* is as high as 0.983, as shown in Fig. 12 Show. Through the *F1*-Confidence curve, the *F1* score changes of the five abrasive grains can be seen respectively. The highest score of all classes is 0.93, and the lowest score is 0.410, as shown in (Fig. 13).

**Fig. 14:**
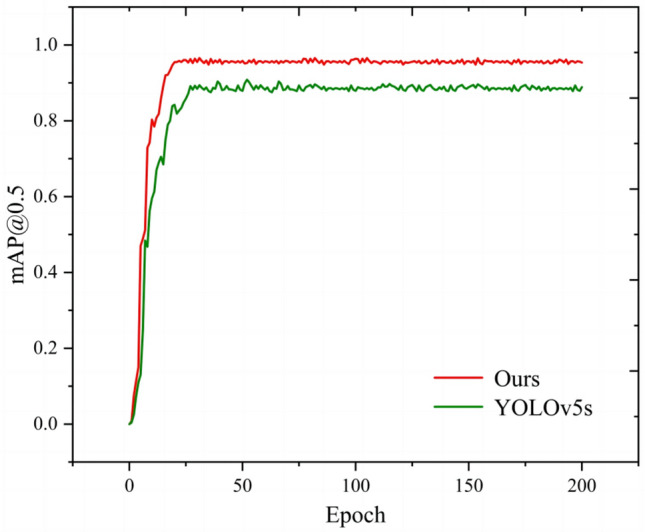
Comparison of the change curves of *mAP@0.5* between TCBGY-Net and YOLOv5s.

In addition, comparing TCBGY-Net with YOLOv5s, the change trend of the *mAP@0.5* curve is shown in Fig. 14. When YOLOv5s is trained to 27 epochs, *mAP@0.5* rises to about 0.88 and then tends to stabilize; while the algorithm in this paper is trained to 18 epochs, *mAP@0.5* rises to about 0.98 and then tends to be stable. Both algorithms are well trained without overfitting or underfitting. Compared with YOLOv5s, the algorithm in this paper has achieved better average detection precision, which verifies the effectiveness of the improvement measures proposed in this paper.

### Ablation experiment

#### Ablation experiments with different attention mechanisms

In order to verify the effect of introducing different attention mechanisms in the C3 module of the neck network^32^, this paper conducted an ablation experiment, respectively introducing the SE(Squeeze-and-Excitation Networks ^33^), ECAM (Enhanced Channel Attention Module, ^34^), CA(Coordinate Attention, ^35^), GA(Ghost Attention, 2022) and CBAM attention mechanisms, and comparing the experimental results, as shown in (Table 3). The experimental results show that after introducing the CBAM attention mechanism in the C3 module, the highest precision of wear particle detection and recognition can be achieved in complex backgrounds.

**Table 3:** The results of ablation experiments with different attention mechanisms.

Fusion attention mechanism	*P*/%	*R*/%	mAP@0.5/%	FPS/(frame·s^−1^)
C3	88.4	88.0	88.1	42.4
CA + C3	88.9	88.2	89.0	50.5
SE + C3	88.7	88.5	88.7	50.6
ECAM + C3	89.3	89.2	89.4	51.2
CBAM + C3	90.9	90.1	90.7	52.8
GA + C3	89.7	89.4	89.6	52.9

**Fig. 15:**
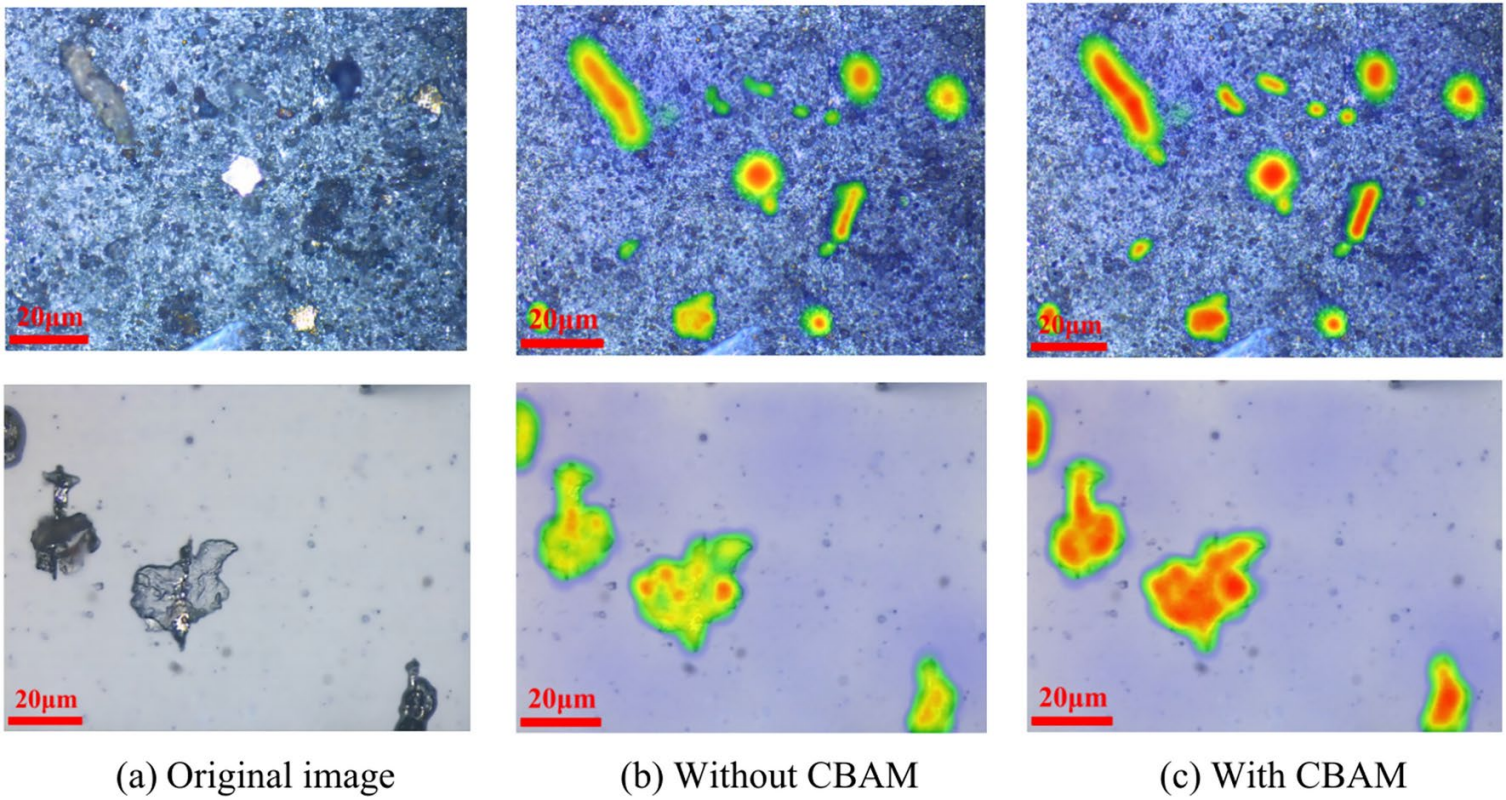
CAM visualization before and after improvement.

In order to further verify the wear particle detection performance in the complex background after fusing the CBAM attention mechanism in the C3 module of the neck network, the CAM heat map visualization analysis was performed on the ferrography wear particle image. Figure 15 is the visual result of the CAM heat map in the environment, and the red area is the salient area that the network model focuses on, and the darker the color, the higher the salient degree^36^. It can be seen from Fig. 15 that there are negative sample areas similar to abrasive grains in the background, and these areas are darker, indicating that they are also focused on by the network model, and are easily false drop as wear particles, and after the CBAM attention is introduced in the C3 module after the mechanism, the significance of the wear particle to be detected can be enhanced, and the attention of features in irrelevant areas can be reduced.

#### Ablation experiments of different improved modules

In order to better verify the effectiveness of the improvement of the YOLOv5s algorithm in this paper, we use different improved modules to conduct ablation experiments, and the experimental results are summarized in (Table 4).

**Table 4:** Ablation experiment results of different improved modules.

Method	Add improvement modules	*P*/%	*R*/%	mAP@0.5/%	FPS/(frame·*S*^*-1*^)	AP_small (%)	FLOPs (G)
I	YOLOv5s	88.4	88.0	88.1	42.4	82.5	16.5
II	YOLOv5s + C3TR	91.9	91.4	91.2	59.2	84.0	17.0
III	YOLOv5s + C3TR + CBAMC3 × 1	93.6	92.4	92.7	71.8	87.1	17.5
IV	YOLOv5s + C3TR + CBAMC3 × 1 + BiFPN	96.2	94.9	95.6	78.5	92.5	18.0
V	YOLOv5s + C3TR + CBAMC3 × 1 + BiFPN + GCBS	97.7	95.4	98.3	89.2	94.6	13.0
VI	YOLOv5s + C3TR + BiFPN	94.5	93.2	94.0	73.0	90.2	17.2
VII	YOLOv5s + C3TR + GCBS	95.0	93.5	94.3	83.5	88.5	13.1
VIII	YOLOv5s + CBAMC3 × 1 + BiFPN	95.6	93.8	94.8	75.2	91.8	16.5
IX	YOLOv5s + CBAMC3 × 1 + GCBS	94.3	92.7	93.4	82.5	88.0	13.8
X	YOLOv5s + BiFPN + GCBS	96.4	94.3	96.0	85.0	93.0	12.9

Based on the analysis of Table 4, several key findings can be observed. After adding the improved C3TR module to the original YOLOv5s model (Model II), the precision rate and recall rate increased by 3.96 and 3.86%, respectively, with the *mAP@0.5* increasing by 3.52%, and the number of frames transmitted per second increasing by 16.8FPS. This not only improved these key metrics but also strengthened the global feature information extraction ability of the backbone network. With the addition of the CBAMC3 × 1 module (Model III), the significance of wear particle detection was enhanced, particularly for small object detection, resulting in an additional precision improvement of 1.85%, an increase in recall by 1.09%, and a rise in FPS by 12.6. When the BiFPN structure was introduced (Model IV), precision and recall rates further increased by 2.78 and 2.71%, respectively, with the *mAP@0.5* rising by 3.13% and FPS increasing by 6.7FPS. This improvement was attributed to the efficient multi-scale feature fusion of BiFPN, which significantly improved small object detection.

Further examination of the other combinations reveals additional insights. In Model VI (YOLOv5s + C3TR + BiFPN), although CBAMC3 × 1 was removed, BiFPN and C3TR together provided strong results, with an precision of 94.5% and an *AP_small* of 90.2%, showing BiFPN’s significant contribution to small object detection. In contrast, Model VII (YOLOv5s + C3TR + GCBS) demonstrated a trade-off between speed and precision, with FPS increasing to 83.5 but *AP_small* decreasing to 88.5%, indicating that while GCBS accelerates computation, it is less effective in improving small object detection compared to BiFPN. Model VIII showed an improvement in both precision and small object detection (*AP_small* = 91.8%) compared to Model VI (YOLOv5s + C3TR + BiFPN). However, the FPS decreased from 73.0 to 75.2, which can be attributed to the additional computational overhead introduced by the CBAMC3 × 1 module. Model IX highlighted the importance of BiFPN, as the exclusion of BiFPN led to reduced performance across multiple metrics, including a lower *AP_small* of 88.0%. Model X (YOLOv5s + BiFPN + GCBS) demonstrated strong performance in both speed (FPS = 85.0) and small object detection (*AP_small* = 93.0%), showing that BiFPN and GCBS together offer an efficient balance of multi-scale feature fusion and lightweight computation.

Finally, in Model V (YOLOv5s + C3TR + CBAMC3 × 1 + BiFPN + GCBS), the combination of all these modules achieved the best overall performance, with the highest precision of 97.7%, recall of 95.4%, mAP@0.5 of 98.3%, and FPS of 89.2, along with an outstanding AP_small of 94.6%. To highlight the efficiency of TCBGY-Net, the Ghost module reduces FLOPs by 21.2% compared to YOLOv5s while maintaining superior detection performance.This highlights the synergy between C3TR, CBAM, BiFPN, and GCBS, which together provide both enhanced feature representation and efficient computation. In our experiments, the simultaneous increase in both detection speed and accuracy can be attributed to the careful integration of these modules, which work synergistically to improve detection performance without significantly increasing computational complexity. The enhanced feature representation from GCBS, BiFPN, CBAM, and the improved C3TR modules leads to a balanced improvement in both speed and accuracy, making Model V the optimal combination for detecting small targets with high precision and speed.

#### Performance comparison of similar detection models

**Fig. 16:**
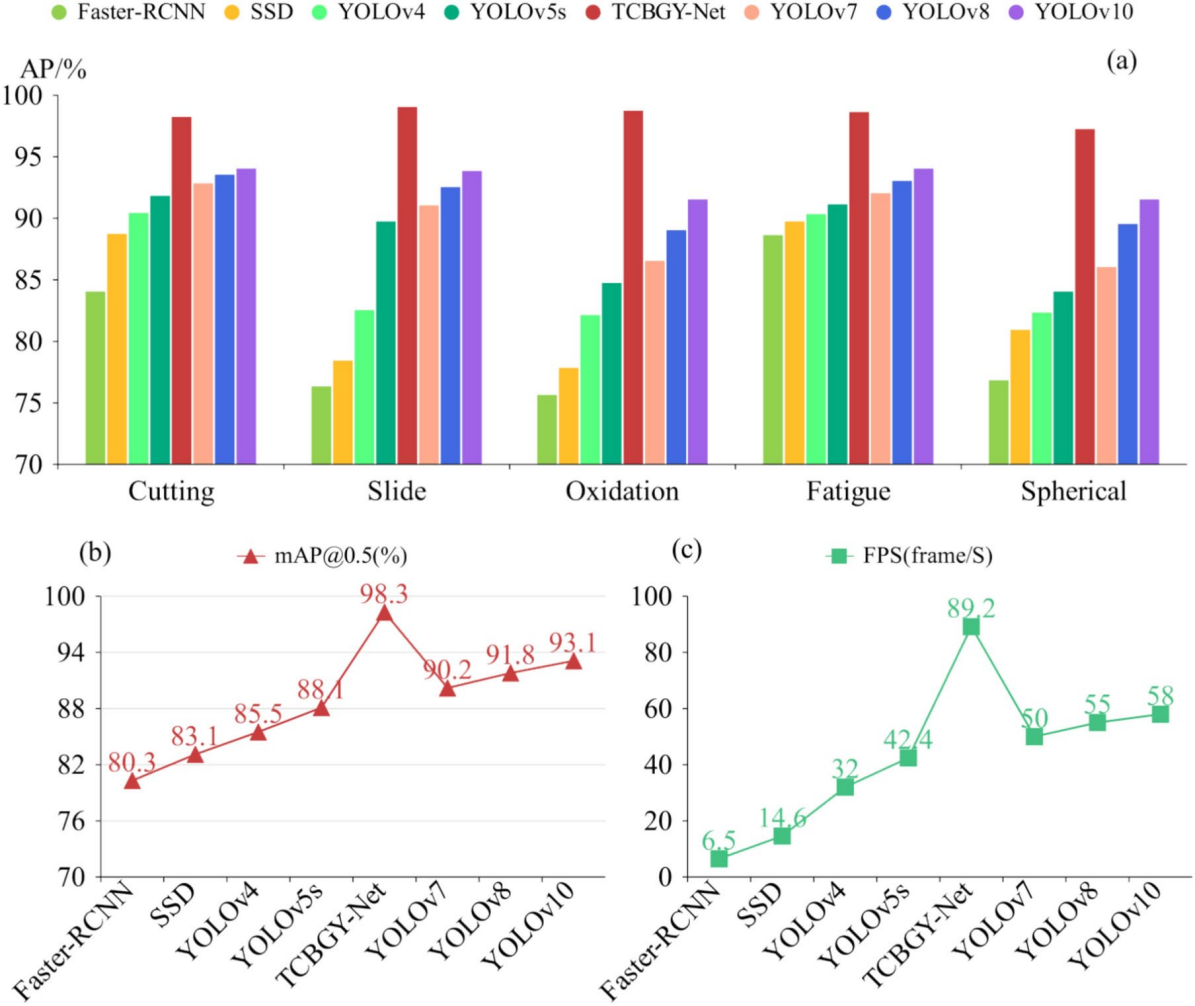
Comparison results of different algorithms: (**a**) illustrates the precision of five types of wear particles identified by different algorithms; (**b**) shows the *mAP@0.5* performance comparison across the algorithms; and (**c**) depicts the detection speed of each algorithm.

To objectively evaluate the performance of each object detection algorithm and verify the advantages of the TCBGY-Net algorithm, we used the same dataset and training parameters across different algorithms. The experimental results are presented in (Fig. 16). Compared to the *mAP@0.5* of Faster RCNN, SSD, YOLOv4, YOLOv5s, YOLOv7^37^, YOLOv8^38^, and YOLOv10^39^, the TCBGY-Net algorithm shows improvements of 22.4, 18.3, 12.8, 10.2, 9.0, 7.1, and 5.6%, respectively. Furthermore, TCBGY-Net achieves the highest precision when detecting five types of wear particles, with a *mAP@0.5* of up to 98.3%. In addition, the detection speed (FPS) of TCBGY-Net is also significantly higher than other models, making it more suitable for wear particle detection and identification tasks.

**Fig. 17:**
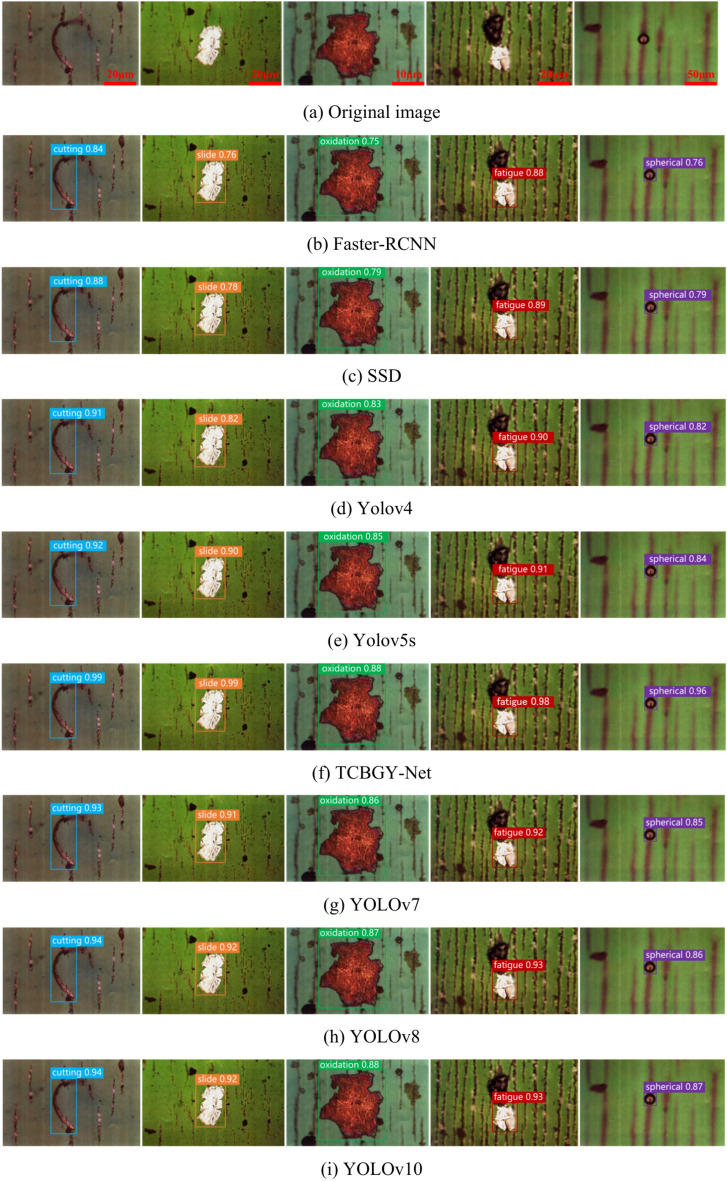
Comparison of single-target wear particle recognition effects of mainstream algorithms.

**Fig. 18:**
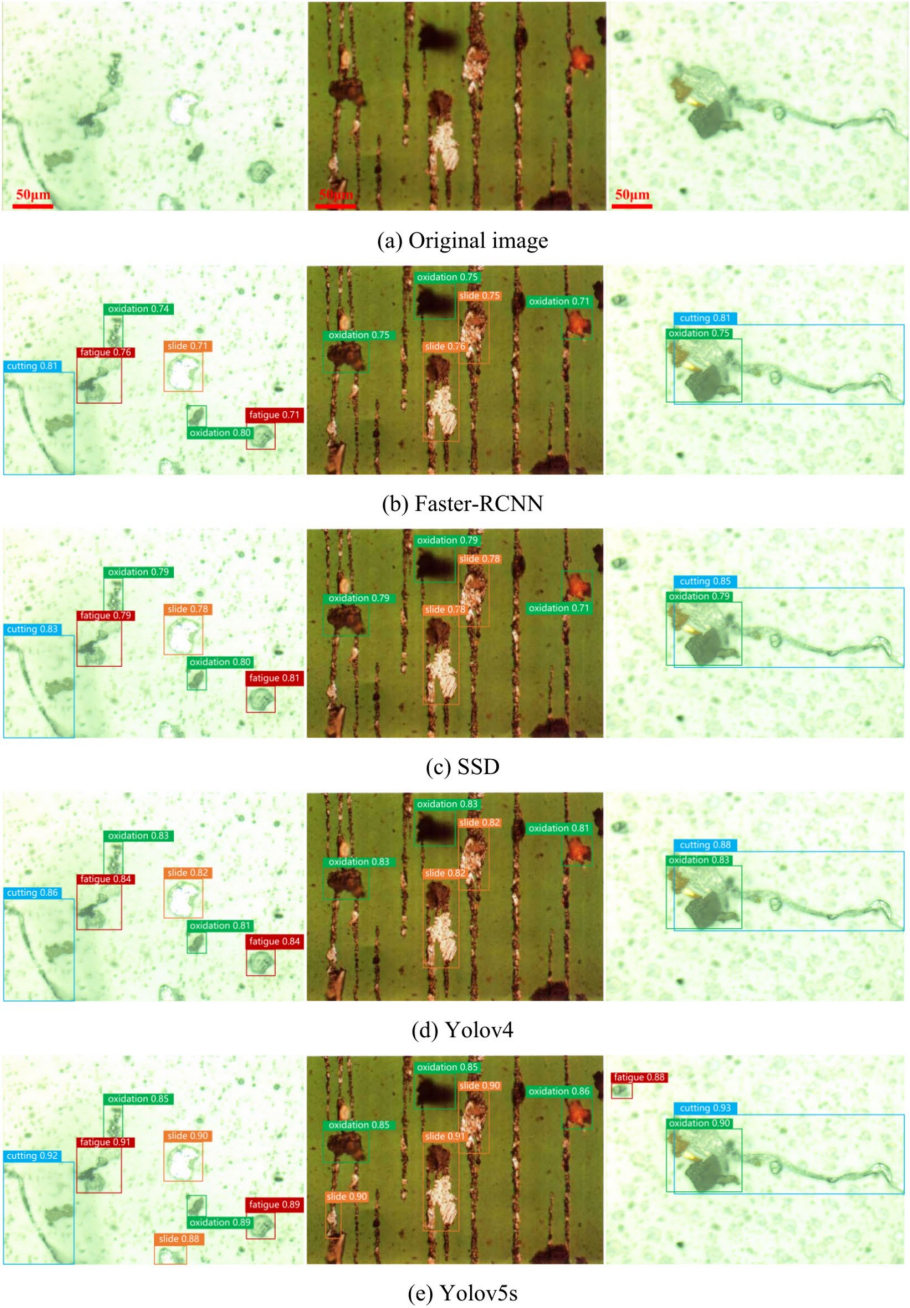

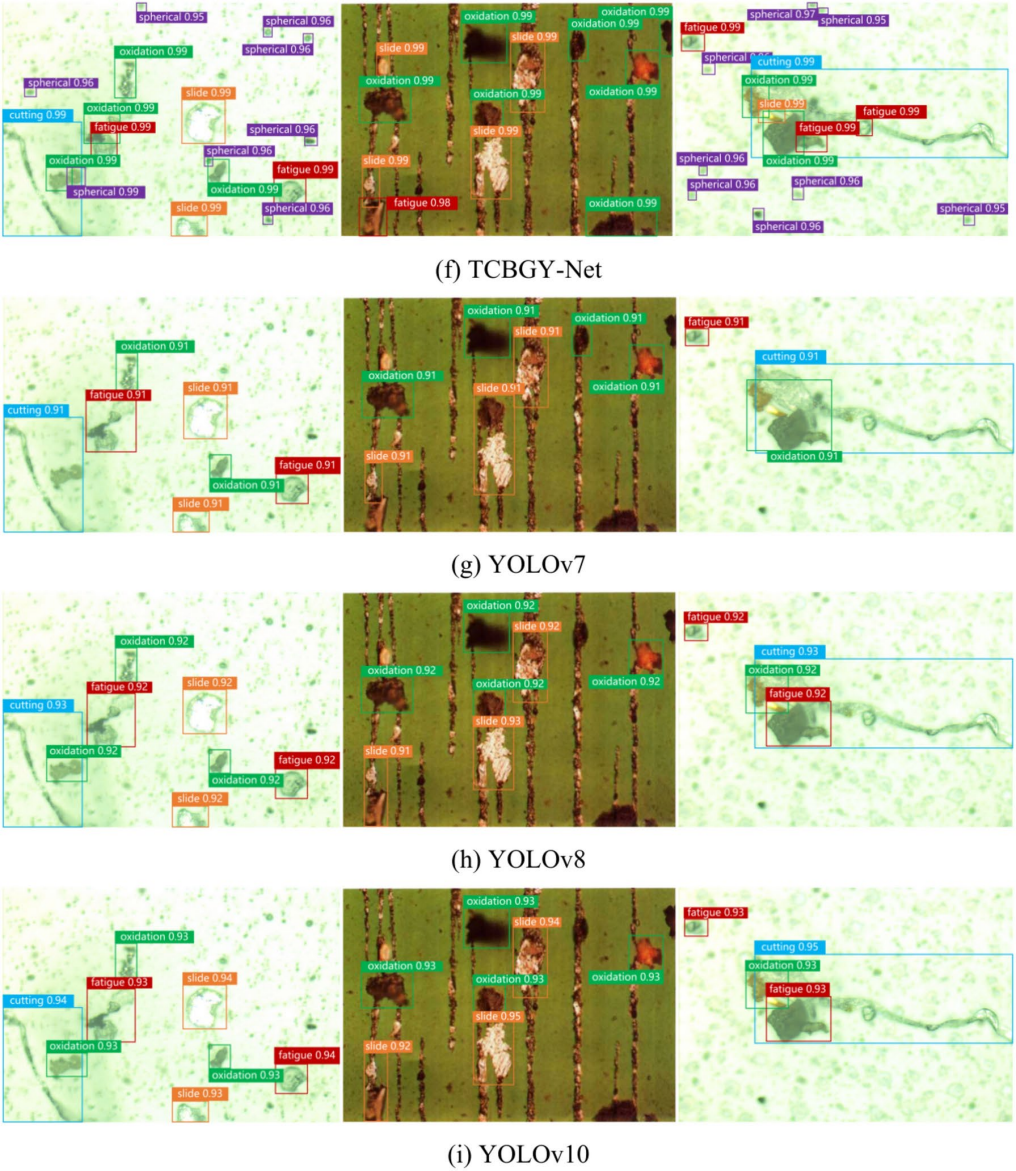
Comparison of multi-objective wear particle recognition effects of mainstream algorithms.

In order to demonstrate the advantages of the TCBGY-Net algorithm more clearly, select representative single-target and multi-target wear particle pictures, and compare them with Faster RCNN, SSD, YOLOv4, and YOLOv5s, YOLOv7, YOLOv8, YOLOv10. Figure 17 shows the comparison of detection results of single-target wear particles in different algorithms, and Fig. 18 shows the comparison of detection results of multi-target wear particles in different algorithms.

Figure 17a presents the original image of a single-target wear particle, while Fig. 18a displays the original image of multiple-target wear particles. Figures 17g–i and 18g–i illustrate the recognition effects of YOLO series algorithms (YOLOv7, YOLOv8, YOLOv10) and demonstrate their gradual improvements compared to earlier models like Faster RCNN, SSD, YOLOv4, and YOLOv5s.

In Fig. 17, it is evident that while all mainstream algorithms can identify single-target wear particles, TCBGY-Net achieves the highest precision and consistently outperforms the YOLO series in accurately detecting each type of wear particle. For example, the precision for recognizing “cutting” and “severe slide” particles reaches 99.0% and 98.7% respectively, surpassing the performance of the other algorithms which often showed difficulties in effectively extracting the complete features of the wear particles. TCBGY-Net delivers not only higher recognition accuracy but also more reliable bounding boxes, leading to precise localization even in challenging conditions.

In Fig. 18, which focuses on multi-objective wear particle recognition, the advantages of TCBGY-Net become even more evident. In complex scenes involving multiple particles, Faster RCNN and SSD exhibit significant limitations in detecting wear particles, particularly under conditions of overlapping, complex backgrounds, and similar visual characteristics, as shown in (Figs. 18b,c). YOLOv4 and YOLOv5s perform somewhat better but still face challenges when dealing with smaller or overlapping particles, as shown in (Fig. 18d–e). Although the YOLOv7, YOLOv8, and YOLOv10 models improved detection of small targets and overlapping particles, TCBGY-Net provides the best performance across the board. As illustrated in Fig. 18f, TCBGY-Net can precisely identify overlapping, small, and visually similar particles with an accuracy significantly higher than the other models, reaching 99.0% for “severe sliding” wear particles and 98.7% for “oxidation” wear particles.

Compared to the YOLO series algorithms, TCBGY-Net demonstrates its clear superiority by consistently delivering higher detection precision and recall, particularly for small and overlapping wear particles in complex backgrounds. The integration of the C3TR module for enhanced global feature extraction, the improved BiFPN for efficient multi-scale feature fusion, and the CBAMC3 × 1 attention mechanism significantly improves the model’s ability to focus on critical features while suppressing irrelevant information. This combination allows TCBGY-Net to achieve optimal results, with notable improvements in detecting small targets and accurately recognizing overlapping wear particles, where other models like YOLOv10 may still struggle.

Furthermore, TCBGY-Net not only excels in accuracy but also maintains competitive detection speed, which makes it particularly suitable for real-time wear particle identification tasks in industrial applications. This superior performance is evident in both single-target and multi-objective detection scenarios, highlighting the robustness and reliability of TCBGY-Net compared to existing mainstream algorithms.

### Evaluation of generalization capability

**Fig. 19:**
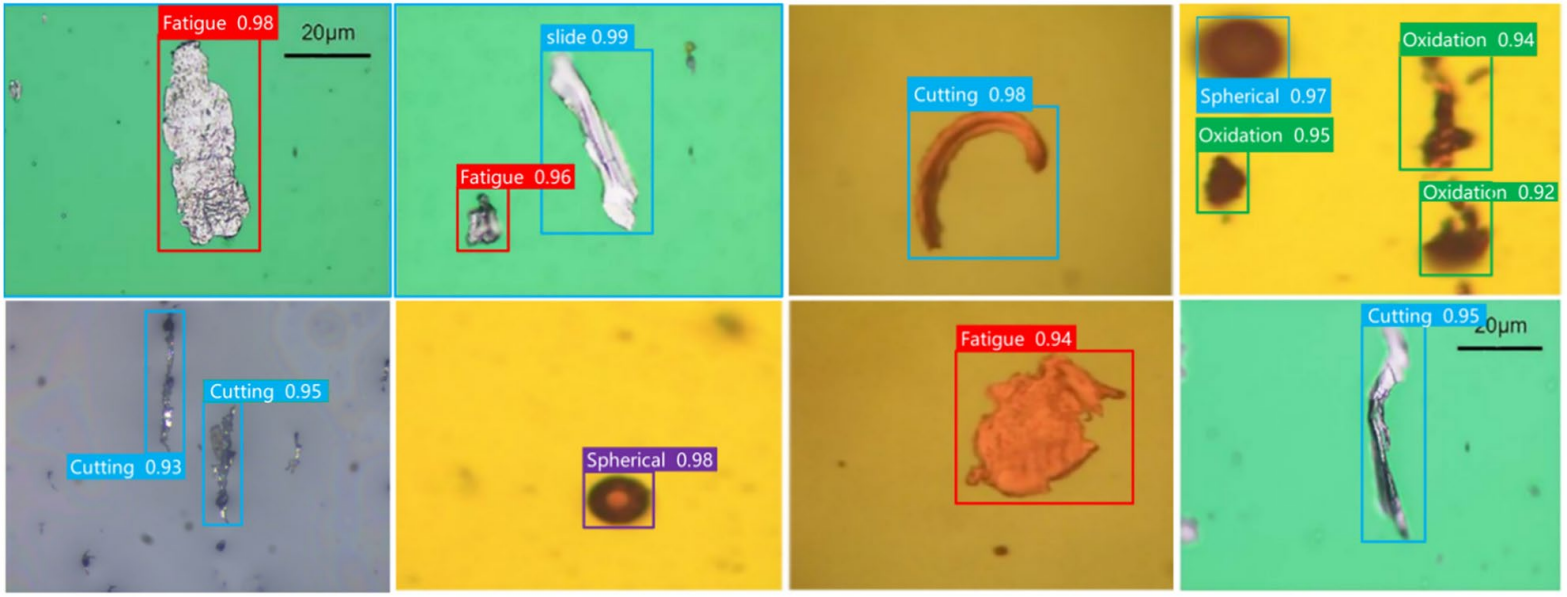
Visualization of object detection results in generalization capability evaluation.

To evaluate the generalization capability of the TCBGY-Net model, we conducted detection and recognition tests on ferrography wear particle images under different and unfamiliar conditions. Specifically, we used the test set from our dataset along with cropped ferrography wear particle images from references^40^ and^41^ as new test sets. These cropped images are characterized by low resolution, blurriness, coexistence of small and multiple wear particles, and generally poor image quality, making them well-suited for evaluating the generalization performance of TCBGY-Net in complex environments. The experimental results are presented in (Tables 5 and Fig. 19).

**Table 5:** Results of generalization capability evaluation experiments.

Type	*P*/%	*R*/%	*AP*/%	mAP@0.5/%	*F1*/%
Fatigue	97.11	95.43	96.11	94.58	96.26
Cutting	97.35	95.22	96.24	94.41	96.27
Slide	97.55	95.34	96.78	94.87	96.43
Oxidation	97.13	95.76	96.14	94.48	96.44
Spherical	97.23	95.54	96.51	94.66	96.38

Despite the low resolution of the cropped images, TCBGY-Net was still able to accurately recognize the wear particles in the ferrography images, achieving over 95% precision for each type of particle. This result verifies the strong generalization capability of the TCBGY-Net algorithm for detecting and recognizing wear particles in ferrography images, demonstrating its applicability under various conditions and highlighting its robustness and practicality.

## Conclusion

The TCBGY-Net algorithm proposed in this paper improves ferrography wear detection by addressing issues with small wear particles, overlapping and similar particles, and complex backgrounds. Compared to the original YOLOv5s model, the enhanced TCBGY-Net achieved superior detection results on a dataset composed of 5 types of wear particles. The average detection precision increased by 10.2%, reaching 98.3%, and the detection speed is as high as 89.2FPS, which fully demonstrates the model’s advantages in terms of precision and speed. A good balance is maintained between them. Through comparative experiments, it is intuitively shown that the TCBGY-Net algorithm has stronger robustness and higher detection precision in complex backgrounds. It is also more suitable for fast, concise, and high-precision detection of ferrography wear particle images. Furthermore, it has great potential for engineering applications that require real-time online detection. In future research, TCBGY-Net will be integrated into the ship’s intelligent engine room system to optimize a deep learning model with higher precision, smaller size, faster speed, and stronger generalization ability. This will make it more suitable for online oil detection systems and enable fault diagnosis and predictive maintenance of ship power plants.

## Data Availability

The data that support the findings of this study are available from the corresponding author upon reasonable request. For experts and scholars who need the ferrography wear particle image dataset, please contact helei0014@stu.shmtu.edu.cn via email. We will provide the full dataset free of charge for research purposes.
